# Mendelian susceptibility to mycobacterial disease: IFN-γ-driven immunity collapse underlies heterogeneous infections

**DOI:** 10.3389/fimmu.2026.1781297

**Published:** 2026-05-08

**Authors:** Mengqing Qian, Jingyu Zhou, Qinhua Zhou, Qingla Zeng, Lingyun Shao, Wenhong Zhang, Qiaoling Ruan

**Affiliations:** 1 Shanghai Key Laboratory of Infectious Diseases and Biosafety Emergency Response, National Medical Center for Infectious Diseases, Department of Infectious Diseases, Huashan Hospital, Fudan University, Shanghai, China; 2 Department of clinical immunology, Children’s hospital of Fudan University, Fudan University, Shanghai, China; 3 Shanghai Sci-Tech Inno Center for Infection & Immunity, Shanghai, China

**Keywords:** IFN-γ, inborn errors of immunity, infection, mendelian susceptibility to mycobacterial disease (MSMD), mycobacterial infection

## Abstract

Mendelian susceptibility to mycobacterial disease (MSMD) is a rare inborn error of immunity characterized by heightened susceptibility to low-virulence non-tuberculous mycobacteria. Despite the widespread application of next-generation sequencing, the molecular etiology of approximately 50% of patients remains elusive. To date, 22 genes have been implicated, all converging on the IL-12/23–IFN-γ circuit, underscoring its non-redundant role in controlling intracellular pathogens. Isolated MSMD is characterized by a selective predisposition to one or more mycobacterial and related infections. But syndromic MSMD’s clinical phenotypes are highly heterogeneous; apart from mycobacterial infections, patients may suffer from viral, bacterial, or fungal diseases, and can additionally manifest auto-inflammation, malignancy, or cutaneous involvement. Current MSMD management mainly hinges on prolonged antimicrobial therapy or align with recombinant human interferon-γ (rhIFN-γ), although allogeneic hematopoietic stem cell transplantation (HSCT) remains the sole curative yet high-risk option; gene editing is still experimental. Priorities are early high-risk identification, targeted intervention and full-process management.

## 1 Introduction

**Figure 1:**
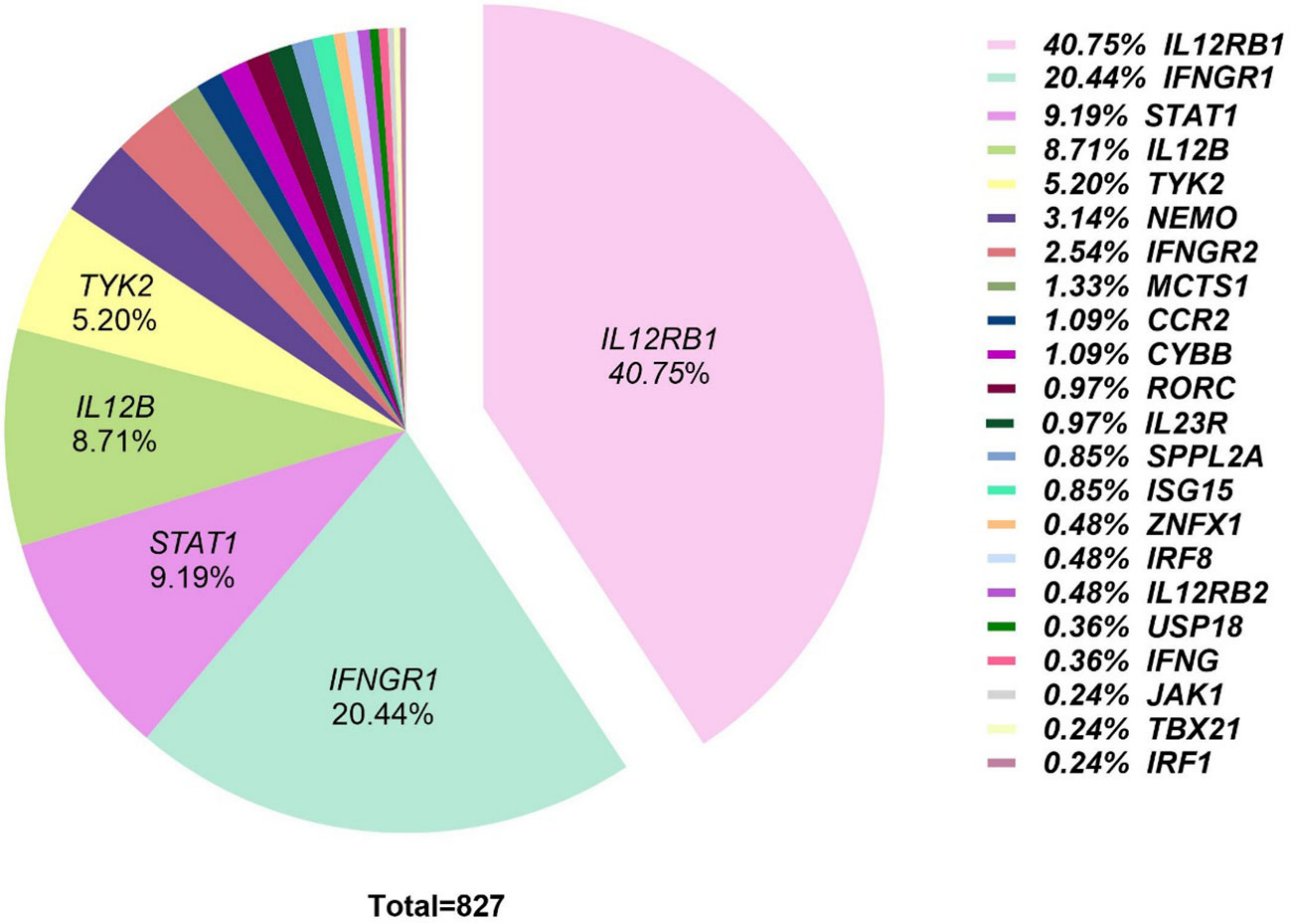
Relative prevalence of mutated genes in 827 genetically defined MSMD patients. The pie chart illustrates the distribution of disease-associated genes among 827 patients. The five most frequently mutated genes are *IL12RB1* (40.75%), *IFNGR1* (20.44%), *STAT1* (9.19%), *IL12B* (8.71%), and *TYK2* (5.20%), collectively accounting for approximately 84% of cases. These five most prevalent genes encode critical components of IFN-γ production (*IL12B, IL12RB1, TYK2*) and cellular response (*IFNGR1, STAT1*) pathways.

**Figure 2:**
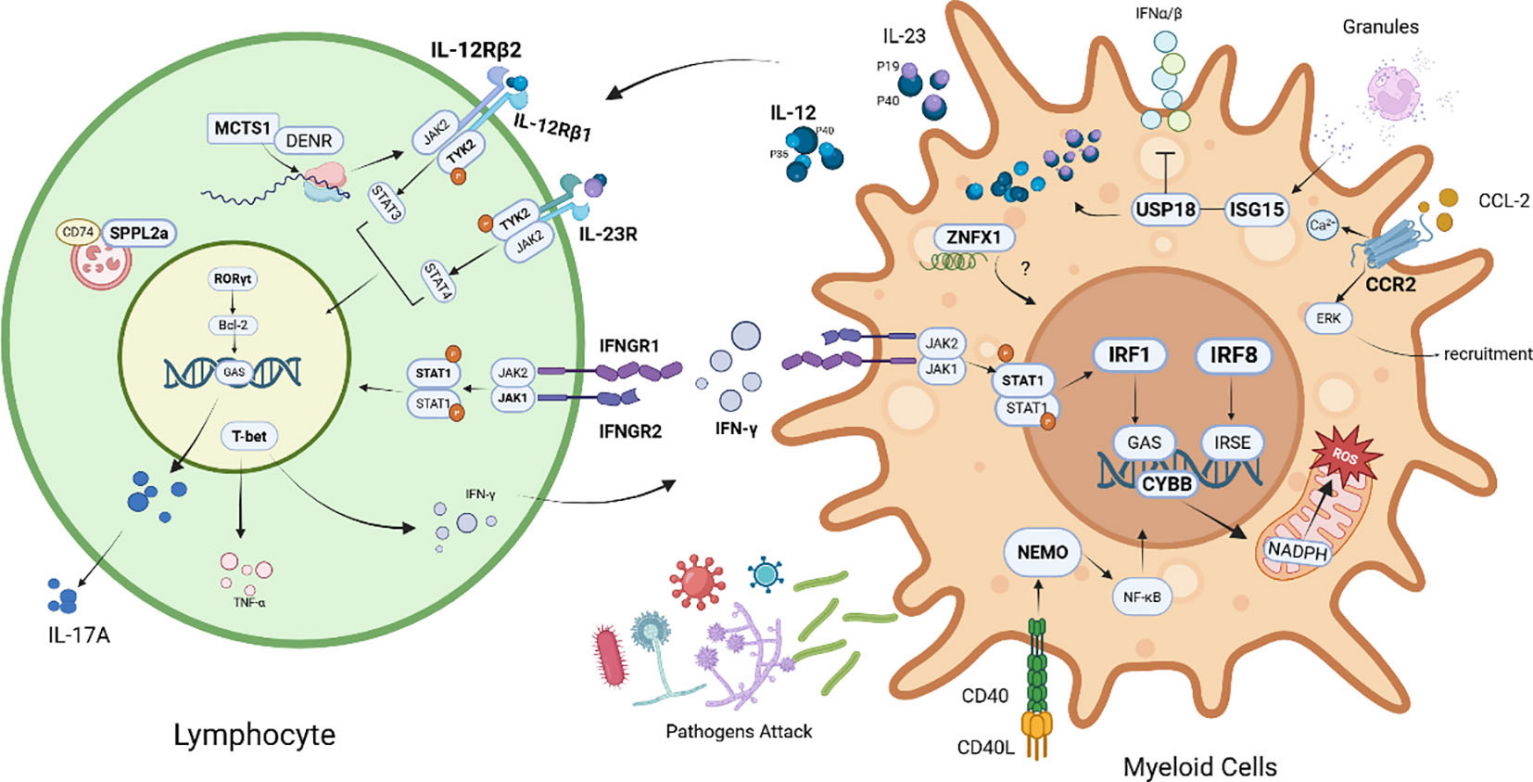
Schematic diagram of IL-12/23-IFN-γ signaling loop defects in the pathogenesis of Mendelian susceptibility to mycobacterial disease. In lymphocytes, IL-12 binds to its receptors (IL-12Rβ1/IL-12Rβ2), activating JAK2/TYK2 kinases, phosphorylating STAT3/STAT4, and subsequently promoting T-bet transcription factor expression, driving IFN-γ production. IFN-γ activates the JAK1/JAK2-STAT1 signaling axis through the IFNγR1/IFNγR2 receptors, forming a positive feedback loop. IL-23 signaling also activates the JAK-STAT pathway via IL-23R, synergistically regulating IFN-γ and IL-17A secretion with RORγt. Furthermore, SPPL2a regulates antigen presentation via CD74, and the MCTS1/DENR complex participates in JAK2 translation regulation, affecting IFN-γ secretion. In myeloid cells, after pathogen invasion, IFN-α/β activates STAT1 via JAK1/JAK2, inducing IRF1/IRF8 transcription factor expression, promoting CYBB (NADPH oxidase gp91phox subunit) transcription, and generating reactive oxygen species (ROS) to kill pathogens. NEMO-mediated NF-κB pathway and CD40-CD40L co-stimulatory signaling participate in inflammation regulation. Molecules such as ZNFX1, USP18, and ISG15 participate in interferon signaling regulation. CCR2 mediates the recruitment of myeloid cells to the site of infection via the ERK pathway, and chemokines such as CCL-2 synergistically participate in granuloma formation. [The bolded text represents the MSMD-related mutant genes that have been reported so far.].

Mendelian susceptibility to mycobacterial disease (MSMD) is a rare inherited immune error that makes individuals susceptible to low-virulence Nontuberculous Mycobacteria (NTM) (1). The inheritance pattern of this disease can be X-linked recessive, autosomal dominant (AD), and autosomal recessive (AR). Currently, 22 genes are known to be associated with MSMD, including 19 autosomal genes (*IL12B, IL12RB1, IL12RB2, IL23R, JAK1, RORC, ISG15, USP18, TYK2, IRF8, SPPL2A, IFNGR1, IFNGR2, IRF1, STAT1, TBX21, IFNG, ZNFX1, CCR2*) and 3 X-linked genes (*CYBB, MCTS1*, and *NEMO*) (**Figure 1**). Variations in 22 genes impair IFN-γ production, cellular responses to this cytokine, or both (**Figure 2**). Despite this, the genetic cause of MSMD is only clearly defined in 50% of patients (2). The clinical manifestations of MSMD can be categorized as either isolated or syndromic. Isolated MSMD is characterized by selective susceptibility to one or more mycobacterial species and related infections; syndromic MSMD is characterized by the combination of mycobacterial infection phenotypes with other clinical phenotypes (3, 4). Defects in *CCR2, ISG15, JAK1, RORC, USP18*, and *ZNFX1* can lead to a syndromic phenotype; deficiencies in *CYBB*(XR-MSMD-2), *IFNG, IFNGR1, IFNGR2, IL12B, IL12RB1, IL12RB2, IL23R, IRF1, MCTS1, NEMO, SPPL2A*, and *TBX21* can lead to an isolated phenotype; deficiencies in *IRF8, STAT1*, and *TYK2* can result in both phenotypes. Although the majority of MSMD patients are infected with nontuberculous mycobacteria (NTM), the fundamental pathogenesis of this disorder centers on interferon-γ deficiency, which renders patients susceptible to a broader range of pathogens, particularly intracellular bacteria. Different genetic mutation sites correspond to distinct clinical phenotypes, including variations in pathogen susceptibility patterns.

This review presents a narrative synthesis of the expanding genetic landscape of MSMD, establishing a conceptual framework. We integrate findings from 22 disease-associated genes, systematically mapping their distinctive pathogen susceptibility profiles to illustrate how defects at discrete molecular nodes generate the phenotypic continuum from isolated mycobacterial susceptibility to syndromic multisystem disease. This narrative review synthesizes published data up to February 2026, integrating findings from recent large-scale cohort studies and case reports to provide an updated genetic landscape of MSMD. We also critically juxtapose congenital MSMD with acquired neutralizing anti-IFN-γ autoantibody syndrome (nAIGA), analyzing their mechanistic divergences and functional convergences at the pathway terminus.

## 2 Spectrum characteristics of clinical phenotypes: molecular basis of isolated and syndromic types

Based on the spectrum of accompanying clinical manifestations, MSMD can be classified into isolated, syndromic, and variable forms (3). These phenotypic distinctions reflect the functional hierarchy and pathway-specific effects of genetic defects within the immune network.

Isolated MSMD is characterized by selective susceptibility to mycobacteria, typically without structural abnormalities. This phenotype commonly arises from defects in core components of the IL-12/23–IFN-γ axis (5). Because these genes operate relatively specifically within this signaling loop, primarily affecting cytokine production or receptor signal transduction (6).

Syndromic MSMD, conversely, presents with mycobacterial infection as the central feature, often accompanied by autoimmunity, or structural developmental anomalies (3). The causative genes typically encode pleiotropic regulators or shared components of multiple pathways, frequently exhibiting low functional redundancy (7). This lack of compensatory backup renders specific pathways vulnerable to collapse upon genetic perturbation. At the transcriptional level, *RORC* regulates Th17 and Th1 cell plasticity, leading to abnormal adaptive immune differentiation (8).*CCR2* deficiency causes impaired monocyte migration, while ISG15-USP18 axis dysfunction disrupts protein homeostasis and type I interferon negative feedback (9, 10). These cross-pathway functional defects overlap, collectively forming a multisystemic clinical phenotype centered on mycobacterial susceptibility, accompanied by other clinical phenotypes.

Variable phenotypes reflect how allelic heterogeneity and differences in residual function modify clinical severity (11). Dominant-negative mutations may selectively disrupt the IFN-γ signaling module while preserving other immune functions, yielding isolated MSMD, whereas complete loss-of-function eliminates all molecular activities, breaching system redundancy and producing syndromic disease (7, 12, 13). In contrast, recessive hypomorphic alleles (e.g., *TYK2* P1104A) demonstrate incomplete penetrance through residual kinase activity sufficient to maintain non–IL-12/23 pathway functions, thereby attenuating disease severity (14, 15). Collectively, these mechanistic insights delineate a “genotype-residual function-phenotype” axis, illustrating how the selective or global collapse of immunological modules shapes the diversity of clinical outcomes in mycobacterial infections.

## 3 Pathogenic genes

### 3.1 Isolated forms genes

#### 3.1.1 CYBB

The *CYBB* gene encodes the gp91phox component of the phagocyte-type nicotinamide adenine dinucleotide phosphate (NADPH) oxidase, which is the source of reducing equivalents for the neutrophil respiratory burst oxidase (**Figure 2**) (16).Germline mutations in *CYBB* are the most common cause of chronic granulomatous disease (CGD) (17).All patients with mutations in this gene have been reported to have Bacillus Calmette–Guérin vaccine (BCG), and some have developed true disseminated mycobacterial disease. The mutations discovered so far have been shown to affect the respiratory burst function of monocyte-derived macrophages and EBV-B cells (18). In contrast to the observations in CGD patients, patients with MSMD caused by *CYBB* mutations have only impaired respiratory burst in monocyte-derived macrophages, which is mainly caused by cell-specific impairment of NADPH oxidase assembly. Multipoint linkage analysis in the two pedigrees delineated candidate intervals at Xp11.3-Xp21.1 and Xq25-Xq26.3 (maximum LOD = 2.29), within which hemizygous *CYBB* missense mutations c.692A>C (p.Q231P) and c.532A>C (p.T178P) were subsequently identified. The variants strictly co-segregate with the MSMD phenotype in affected males and with carrier status in obligate females, conforming to X-linked recessive inheritance and indicating a founder effect. The patients have no history of granulomatous disease and present with BCG and *Mycobacterium tuberculosis* infection alone (**Table 1**) (19). Recently, a novel hypomorphic *CYBB* variant (c.998C>A, p.S333Y) was identified in an adult male with disseminated *Mycobacterium avium* complex infection, representing the second report of XR-MSMD caused by *CYBB* missense mutation and the first documented adult-onset case (20) (**Figure 1**). The reported patients have a good prognosis.

**Table 1:** Summary of reported gene inheritance patterns and mutation numbers in MSMD, and infection spectrum.

Gene	Mutation counts	Inheritance	Mycobacterial infection spectrum	Other infection spectrum
*IL12RB1*	106	AR	BCG; *M. occultum, M. gordonae, M. genavense, M. tilburgii, M. triplex, M. simianum*, *M. avium* complex	*Candida* spp.*, Paracoccidioides brasiliensis, Coccidioides* spp.*, Histoplasma capsulatum, Cryptococcus neoformans; Toxoplasma gondii, Leishmania* spp.*; Salmonella* spp.
*IFNGR1*	49	AR/AD	BCG;*M. chelonae, M. fortuitum, M. margaritense, M. smegmatis, M. scrofulaceum, M. abscessus, M. szulgai, M. asiaticum, M. gordonae, M. kansasii*	CMV, HHV-8, RSV, PRV-3, VZV; *Listeria monocytogenes, Salmonella* spp.*, Streptococcus viridans*
*STAT1*	42	AR/AD	BCG;*M. tuberculosis, M. avium, M. genavense, M. szulgai*	HSV-1, CMV, HHV-8, HPIV, RSV;*Aspergillus* spp.
*IFNGR2*	32	AR/AD	BCG;*M. abscessus, M. avium, M. occultum, M. suis, M. simianum, M. bovis, M. elegans*	CMV, EBV
*TYK2*	18	AR	BCG, *M. tuberculosis*	HSV-1, VZV, H1N1, SARS-CoV-2, RSV, EBV, CMV, RV(*Rubella virus*), JCV, *Candida* spp.*, non-typhoidal Salmonella, Staphylococcus aureus*
*ISG15*	15	AR	BCG	*Klebsiella pneumoniae, Candida albicans*
*ZNFX1*	14	XR	BCG, *M. tuberculosis*	IAV, IBV, PIV, RSV, NoV, RV(*Rotavirus*), HHV, AdV, CMV, *Salmonella* spp.*, Candida* spp.*, Klebsiella* spp.*, Nocardia* spp.
*IL12B*	12	AR	BCG	/
*NEMO*	12	XR	*M. avium, M. kansasii*	CMV, HSV*; Streptococcus pneumoniae, Haemophilus influenzae, Staphylococcus aureus, Pseudomonas aeruginosa, Leishmania* spp.
*MCTS1*	8	XR	BCG; *M. abscessus*	/
*IRF8*	5	AR/AD	BCG	SARS-CoV-2, influenza A (H1N1), HRV, EBV, HHV-6, *Mycoplasma* spp.*, Candida* spp.
*CCR2*	4	AR	BCG	/
*IL23R*	3	AR	BCG, *M. tuberculosis*	*Candida* spp.
*RORC*	3	AR	BCG, *M. tuberculosis*	*Candida* spp.
*CYBB*	3	XR	BCG, *M. tuberculosis*, *M. avium*	/
*IFNG*	2	AR	BCG	RSV
*IRF1*	2	AR	BCG, *M. avium, M. genavense, M. mucogenicum, M. heckeshornense*	*Histoplasma capsulatum*
*JAK1*	2	AR	*M. malmoense, M. scrofulaceum, M. gordonae*	*/*
*SPPL2A*	2	AR	BCG	/
*IL12RB2*	1	AR	BCG, *M. tuberculosis*	/
*TBX21*	1	AR	BCG	/
*USP18*	1	AR	BCG	/

BCG, Bacillus Calmette-Guérin; EBV, Epstein-Barr Virus; HSV, Herpes Simplex Virus; HHV, Human Herpesvirus; VZV, Varicella-Zoster Virus; RSV, Respiratory Syncytial Virus; HPIV/PIV,(Human) Parainfluenza Virus; PRV-3, Respirovirus 3; IAV, Influenza A Virus; IBV, Influenza B Virus; H1N1, Hemagglutinin 1 Neuraminidase 1; SARS-CoV-2, Severe Acute Respiratory Syndrome Coronavirus 2;JCV, John Cunningham virus; NoV, Norovirus; PIV, Parainfluenza Virus; HRV, Human Rhinovirus.

#### 3.1.2 IFNG

IFN-γ acts as a macrophage-activating factor in the body, primarily secreted by T lymphocytes and NK cells. It is an essential cytokine for protective immunity against low-virulent mycobacteria, exerting its effects through binding to IFN-γR1 and IFN-γR2 (**Figure 2**) (21). Although IFN-γ is a central factor in the pathogenesis of MSMD, only three patients with complete AR *IFNG* deficiency have been reported, harboring a frameshift mutation: p.T119Ifs4* and a missense mutation: p.F75S (**Figure 1**). The *IFNG* gene is believed to have evolved under enhanced purifying selection, thus preventing the accumulation of deleterious monoallelic amino acid variants (1). The physiological reasons for the different selection patterns at the *IFNG* locus and at the *IFNGR1* and *IFNGR2* loci may be related to IFN-γ immunity. Both chains of the receptor are ubiquitously expressed, whereas IFN-γ expression is tightly regulated. Therefore, heterozygous deleterious mutations at the *IFNG* locus are more poorly tolerated than deleterious mutations at the *IFNGR1* and *IFNGR2* loci. The p.T119Ifs4* mutation results in loss of IFN-γ expression and function, and the patients’ blood T cells and NK cells are unable to produce and secrete detectable IFN-γ. The p.F75S mutation results in the inability of IFN-γ to fold or secrete correctly in recipient cells. The main clinical phenotypes of patients with *IFNG* deficiency are BCG disease and secondary hemophagocytic syndrome (**Table 1**). One of the patients underwent HSCT after antimycobacterial treatment but died 9 days after HSCT. Another patient was initially treated with antimycobacterial drugs and subsequently received HCST and is currently in good condition (1).A 30-month treatment regimen of antimycobacterial treatment combined with recombinant IFN-γ1b was tried for the first time in patients carrying the PF75S mutation, and the patients recovered well after treatment. It is the preferred cytokine replacement therapy for patients with complete *IFNG* deficiency after early detection of AR (22).

#### 3.1.3 IFNGR1

A total of 169 MSMD patients carrying *IFNGR1* mutations have been reported to date, representing approximately 20.4% of all molecularly defined cases (**Figure 1**). Biallelic loss-of-function(LOF) mutations in the *IFNGR1* gene were the first reported mutations causing MSMD, resulting in autosomal recessive complete IFN-γR1 deficiency (23). High levels of IFN-γ are detected in patients’ plasma. The clinical phenotype of patients is characterized by disseminated, life-threatening infections with BCG and/or NTM (including *Mycobacterium chelonae, Mycobacterium fortuitum, Mycobacterium mageritense, Mycobacterium smegmatis*, and *Mycobacterium scrofulaceum*) (**Table 1**). Infections typically begin in early childhood, and the MSMD phenotype is fully manifested by childhood. Other infections caused by viruses such as cytomegalovirus (CMV), human herpesvirus 8 (HHV8), respiratory syncytial virus (RSV), pseudorabies virus-3, and varicella-zoster virus (VZV), as well as bacteria such as *Listeria monocytogenes*, have also been reported. Cases of Salmonella infection have been reported less frequently (24, 25). This defect may predispose to malignancies, with reported malignancies including B-cell lymphoma, pineal germ cell tumors, and Kaposi’s sarcoma (26, 27). Due to the lack of a specific receptor, interferon-γ replacement therapy is not effective. Nevertheless, dose escalation occasionally proves efficacious. There have been reports of the use of recombinant human interferon-α(rhIFN-α) therapy, but clinical responses vary. Exogenous IFN-α therapy may exacerbate mycobacterial disease (28). Antimicrobial therapy should be maintained long-term, and hematopoietic stem cell transplantation (HSCT) is the only known cure (29). However, even with transplants from HLA-matched relatives, a high rate of transplant rejection has been observed, which may be due to the high concentration of IFN-γ in the patients’ plasma (30, 31). Overall, patients with AR complete IFN-γR1 deficiency have a poor prognosis.

Patients with AR partial IFN-γR1 deficiency have detectable IFN-γ in their plasma and mildly impaired cellular immunity (32). The clinical phenotype of patients is milder than that of patients with AR complete IFN-γR1 deficiency. Patients can be infected with BCG and/or NTM (*Mycobacterium avium, Mycobacterium avium* complex*, Mycobacterium abscessus, Mycobacterium szulgai)* (33), and approximately 60% of patients develop osteomyelitis (34). Infections with a variety of other pathogens have also been reported, including bacteria (*Haemophilus influenzae, Klebsiella pneumoniae, Legionella* spp.*, Shigella sonnei, Salmonella* spp.*, Mycoplasma pneumoniae*), viruses (VZV, RSV, molluscum contagiosum virus (MCV), and parasites (*Cryptosporidium* spp.*; Toxoplasma gondii*)(**Table 1**) (25). Antibiotics and IFN-γ therapy are required for several years to contain and ultimately control the infection. Given the relatively mild phenotype of the infection, HSCT is not suitable (25). These patients do not require prophylactic antibiotics. However, patients with partial AR deficiency affecting the initiation codon M1K have severe clinical and immunological phenotypes and a poor prognosis (35).

Mutations that cause AD partial IFN-γR1 deficiency are mainly concentrated in exon 6. Due to the accumulation of truncated IFN-γR1 receptors lacking the recycling domain, a large amount of IFN-γR1 protein can be detected on the cell surface. Despite the presence of the IFN-γR1 protein encoded by the wild-type allele, the IFN-γR1 protein is not suitable for HSCT. The accumulation of nonfunctional IFN-γR1 proteins that lack the STAT1 and JAK1 docking sites for the AR receptors prevents the normal function of the IFN-γR1 dimer through dominant-negative inhibition (36). All mutations have a similar cellular phenotype, characterized by an impaired *in vitro* response to IFN-γ (37). The clinical features of patients are milder than those of patients with complete AR IFN-γR1 deficiency. In general, patients are sensitive to BCG or NTM (*Mycobacterium abscessus, Mycobacterium avium complex, Mycobacterium asiatica, Mycobacterium gordonii, Mycobacterium kansasii, Mycobacterium scrofulae) (*25)(**Table 1**). In most cases, mycobacterial disease can be well controlled with long-term antimicrobial therapy, with or without recombinant IFN-γ therapy (37, 38). IFN-β might be used as a beneficial adjuvant therapy for managing extensive central nervous system mycobacterial infection, especially in patients with *IFNGR1* deficiency (39).

#### 3.1.4 IFNGR2

Complete AR deficiency of IFN-γR2 can be divided into two types: no cell surface expression or residual cell surface expression without function (40, 41). So far, 13 patients from 9 families have been reported (**Figure 1**) (42–45). Among them, the homozygous mutation p.T168N is a glycosylation-induced mutation that leads to impaired cellular response to IFN-γ. The mutation (382-387dup) causes protein misfolding, but this mutation can be rescued by glycosylation inhibitors (40). High levels of IFN-γ can be detected in the plasma of all patients. In healthy heterozygous relatives of patients with AR complete IFN-γR2 deficiency, *IFNGR2* loss-of-function mutations showed a dominant negative effect *in vitro (*46). The clinical manifestations of AR complete IFN-γR2 deficiency are similar to those of complete IFN-γR1 deficiency. The disease presents in early childhood with ill-defined mycobacterium-associated granulomas, often presenting with a severe or even fatal phenotype, with multiple cases of cutaneous squamous cell carcinomas reported (47). The most common pathogens include BCG, *Mycobacterium abscessus, Mycobacterium avium, Mycobacterium fortuitum, Mycobacterium suis, and Mycobacterium simianum* (**Table 1**) (40, 42, 47, 48). Antimicrobial therapy should not be discontinued in these patients, and IFN-γ replacement therapy is not suitable for the treatment of patients without functional receptor defects. HSCT is the only cure for these patients, but the prognosis is poor, attributed to the effect of high IFN-γ concentrations on the transplant response (24, 42, 45, 49).

Some of the reported IFN-γR2 deficiencies include p.S124F, p.R114C, p.G141R, p.G227R, and c.958insT (48, 50–52). Missense mutations in the low-allelic *IFNGR2* gene lead to protein misfolding and abnormal N-glycosylation, which is retained in the endoplasmic reticulum. Cell surface IFN-γR2 expression is weak but not absent (53). A weakened but still present response to IFN-γ is observed in various cell types of the patient. GAF γ-activated sequence (GAS) binding activity is reduced, GAF-dependent target genes can be induced, and IL-12p70 can be produced in small amounts (41, 52). The spectrum of mycobacterial infections consists of BCG, *Mycobacterium abscessus, Mycobacterium bovis, Mycobacterium elegans, Mycobacterium fortuitum, and Mycobacterium simianum* (**Table 1**) (50, 52). The response of patients’ cells to IFN-γ can be rescued using N-glycosylation modifiers, and antimicrobial drugs (with or without combined recombinant IFN-γ replacement therapy) are effective treatments for infection, but HSCT is ineffective (41, 52, 54).

IFN-γR2 deficiency can be inherited in an autosomal dominant manner (**Table 1**). Individuals heterozygous for the *IFNGR2* gene have low levels of IFN-γR2 expression on the cell surface, impaired signal transduction and STAT phosphorylation and GAF-DNA binding in EBV-B cells, and exhibit a pathogenic form of haploinsufficiency (55). This is the lowest penetrance reported primary immunodeficiency disorder caused by haploinsufficiency and autosomal dominant inheritance (56).

#### 3.1.5 IL12B

*IL12B* encodes IL-12p40. IL-12 binds to the receptors IL-12Rβ1 and IL-12Rβ2 on T lymphocytes and NK cells and is a potent inducer of IFN-γ (**Figure 1**) (57). IL12p40 deficiency was the first genetic cytokine deficiency to be discovered. Twelve mutations have been reported in approximately 60 patients, all of which are complete AR deficiencies (**Figure 1**) (58–62). Normal expression of IL12p40 and IL-12p70 is absent in patient cells and constructed cell lines, and IFN-γ levels are significantly reduced (5, 58). Complete AR deficiency of IL-12p40 and IL-12Rβ1 appears to mimic clinical phenotypes. The main clinical features are childhood-onset BCG and Salmonella infections, rare multibacterial co-infections, and rare cases of chronic mucocutaneous candidiasis, nocardiosis, and *Klebsiella pneumoniae* pneumonia (**Table 1**) (58–60, 63). This disorder is very similar to AR complete IL-12Rβ1 deficiency. The differences between the two disorders may reflect the low allelic and ethnic diversity of patients with AR complete IL-12p40 deficiency. Patients receive long-term antimicrobial therapy and recombinant IFN-γ replacement therapy, and most cases are not candidates for HSCT (58).

#### 3.1.6 IL12RB1

The *IL12RB1* gene encodes the IL-12Rβ1 chain, a gp130 protein consisting of an extracellular N-terminal immunoglobulin-like domain, a transmembrane domain, and an intracellular domain. The association of IL-12Rβ1 and IL-12Rβ2 is required for high-affinity IL-12 binding and signaling. IL-12Rβ1 also cooperates with IL-23R to recognize IL-23 dimers formed by IL-12p40 and p19(**Figure 2**). Functional IL-12 receptors are primarily expressed on activated T cells and NK cells (64). Complete AR *IL12RB1* deficiency is the most common cause of MSMD (**Figure 1**) (65). Most mutant alleles are loss-of-function usually with absent receptor expression, and more rarely with surface expression of a non-functional receptor. All patients with complete AR *IL12RB1* deficiency tested were unresponsive to IL-12 and IL-23 stimulation, and all patients produced low levels of IFN-γ (66, 67).The clinical phenotype of complete AR *IL12RB1* deficiency varies widely, ranging from death in early infancy to asymptomatic adulthood, with varying disease durations (67). Mycobacterium infections are the most common infections in these patients (BCG, *Mycobacterium avium* complex*, Mycobacterium fortuitum, Mycobacterium genavense, Mycobacterium gordonii, Mycobacterium tilburgii, Mycobacterium chelonae,Mycobacterium simiae*) (**Table 1**) (68–70). These patients have impaired immunity to primary infection with mycobacteria, but their immunity to latent or secondary mycobacterial infections appears to be intact (71). Fungal diseases such as *Candida albicans, Paraspora brasiliensis, Coccidioides immitis, Histoplasma capsulatum, Cryptococcus neoformans*, and parasitic diseases (*Toxoplasma gondii* and *Leishmania*) have also been reported (**Table 1**) (72–76). Vasculitis is a rare clinical phenotype that is typically associated with Salmonella infection (77–79). This variant can also coexist with other clinical phenotypes such as hypogammaglobulinemia, α1-antitrypsin deficiency, ataxia-telangiectasia, neurofibromatosis, and thrombophilia (67, 80–83). Complete AR *IL12RB1* deficiency shows incomplete penetrance, with a penetrance of 0.64 at 5 years of age increasing to 0.79 by 20 years of age (71). The prognosis of this immunodeficiency is variable. Given the incomplete penetrance of this disease, healthy siblings of the proband should be examined to exclude the disease (84). In addition to subcutaneous IFN-γ, patients should receive long-term aggressive antimycobacterial therapy, and abdominal surgery may be used to remove the spleen and/or mesenteric lesions (67). Salmonellosis should also be treated with antimicrobial drugs and IFN-γ replacement therapy, which often improves the vasculitic lesions. If salmonellosis recurs, antimicrobial prophylaxis should be considered (79, 82). HSCT is not indicated, but the overall mortality rate of 26% suggests that this option may be considered in some cases (85).

#### 3.1.7 IL12RB2

IL-12Rβ1 and IL-12Rβ2 heterodimers form the receptor for IL-12 (**Figure 2**). *IL-12RB2* deficiency has been previously reported to cause autoimmune diseases (86). In 2018, Martinez-Barricarte et al. first discovered three patients in a Turkish consanguineous family carrying the homozygous p.Q138* mutation in the gene encoding the IL-12Rβ2 subunit, inherited in a complete AR deficiency pattern (**Figure 1**) (87). Extracellular IL-12Rβ2 expression could not be detected in the cell lines constructed *in vitro*, STAT4 phosphorylation was abnormal after IL-12 stimulation, and the response to IFN-α was normal. Patients with IL-12Rβ2 deficiency had a low frequency of memory Th1 cells, while their Th17 and Th2 cell levels were slightly low or normal. During *in vitro* differentiation, IFN-γ production was abolished under Th1 conditions, which is consistent with reports of IL-12Rβ1 deficiency but contrary to IL-23R deficiency (discussed below). The clinical phenotype is limited to mycobacterial susceptibility, including disseminated tuberculosis and disseminated BCG, with incomplete penetrance typical of MSMD, and the absence of the chronic mucocutaneous candidiasis phenotype reported in patients with *IL-12RB1* deficiency (**Table 1**). The identification and study of families with complete AR IL-12Rβ2 deficiency suggest that IL-12Rβ2 is essential for anti-mycobacterial immunity in a minority of patients.

#### 3.1.8 IL-23R

The binding of IL-23R to its specific ligand IL-23 initiates a series of specific signals by regulating the characteristics and behavior of immune cells (**Figure 2**). It is particularly important for the regulation of helper T cells 17 (Th17) (88). Three mutations have been reported in 8 patients so far (**Figure 1**). The mutations reduce protein expression and produce abnormal N-glycosylation, and abnormal IL-23-induced STAT3 phosphorylation, resulting in abnormal IL-23 response. Patients have reduced MAIT cell levels, abnormally low memory Th1 frequency, and reduced IFN-γ levels after stimulation of mycobacterium-specific CD4^+^ T cells. The scarcity of IL-12Rβ2 or IL-23R-deficient MSMD patients is not because loss-of-function variants at these sites are less rare than those at the IL12RB1 site. Rather, this is due to the low clinical penetrance of MSMD (estimated to be approximately 0.5%), which is consistent with the redundancy of IFN-γ-producing lymphocyte subsets and the partially overlapping roles of IL-12 and IL-23 in this process (89). Overall, IL-12 and IL-23, acting alone or in concert, are required to maintain optimal IFN-γ-dependent immunity.

#### 3.1.9 IRF1

Interferon regulatory factor 1 (IRF1) is a transcription factor that acts downstream of STAT1 and whose expression is mediated by IFN-γ in a GAF-dependent manner. After production, IRF1 protein enters the cell nucleus and binds to promoters containing interferon-stimulated gene element motifs (**Figure 2**) (90). To date, two unrelated patients carrying p. R129∗ and p. Q35∗ have been reported, showing complete AR deficiency (**Figure 1**). IRF1 p. Q35∗ and p. R129∗ mutants result in impaired IRF1 protein expression, and IRF1-deficient mononuclear macrophages are unable to normally control mycobacteria or related macrophage-derived pathogens under IFN-γ stimulation. The immune system defects observed in patients include impaired development of NK cells and naive αβ CD8^+^T cells, as well as impaired IFN-γ production by residual NK cells and γδ2^+^T cells. IRF1 deficiency impairs the development of myeloid dendritic cells, mainly cDC1, and the number of cDC1 in the blood of IRF1-deficient patients is very low. IRF1 is crucial for myeloid cell maturation, though its mechanism is currently unclear and may involve interaction with IRF8. Two patients experienced adverse reactions to BCG vaccination at birth, subsequently developing recurrent or disseminated disease caused by NTM, and presented with early-onset, multi-life-threatening illnesses caused by related macrophage-derived pathogens (**Table 1**). Early-stage *Mycobacterium avium* infection may be due to IFN-γ deficiency, while infection at an older age may be due to the production of IFN-γ autoantibodies, and the clinical penetrance of this deficiency may be complete. Both patients are generally well with regular anti-infective treatment (7, 91).

#### 3.1.10 MCTS1

*MCTS*1 encodes malignant T-cell-amplified sequence 1 (MCTS1), a 181-amino acid polypeptide composed of an unknown functional domain and an RNA binding domain. MCTS1 and DENR (density-regulated reactivation and release factor) act constitutively and are expressed in all cells of the human body. The complex is indispensable for ribosome recycling and translation reactivation (**Figure 2**) (92). The first case of MSMD caused by XR complete *MCTS1* deficiency was reported in 2023, and subsequently, novel pathogenic mutations were identified in unrelated families in four different countries. Reported mutations include early stop codon mutations (p.R72*, p.L170*), frameshift deletions (p.P77Afs*, p.E60K5fs*), and large deletions (p.K4_C55delinsN, p.A133_K181del), all of which are loss of expression (LOE) or loss of function (LOF) variants (93, 94). Furthermore, the p.W175* variant has been confirmed as a sub-effective allele with reduced function when overexpressed, leading to partial XR *MCTS1* deficiency (**Figure 1**) (94). *MCTS1* deficiency selectively inhibits the complete translation of JAK2 mRNA by impairing ribosome recycling. Diminished JAK2 expression disrupts IL-23 signaling specifically within innate-like T cell subsets (Vδ2^+^ γδ T and MAIT cells), leading to defective IFN-γ generation. Notably, this functional deficit is not due to the absence or reduction of specific leukocyte subsets, but rather to an intrinsic cellular signal transduction disorder. In terms of clinical phenotype, complete *MCTS1* deficiency primarily manifests as life-threatening mycobacterial infection following BCG vaccination, which can be multifocal or disseminated and maybe complicated by osteomyelitis. Furthermore, unvaccinated patients may develop *Mycobacterium abscessus* infection. Partial deficiencies may present with a milder phenotype of susceptibility to infection (94). Patients with *MCTS1* deficiency have a relatively better prognosis, and most can survive after anti-infective treatment (93, 94).

#### 3.1.11 NEMO

*NEMO* encodes a regulatory subunit of the Iκβ kinase complex, which is required for the activation of nuclear factor-κβ, which has multiple functions, including regulation of immune and inflammatory responses, osteoclast development, skin epidermal growth, and maintenance of vascular architecture (**Figure 2**) (95). *NEMO* mutations result in distinct clinical and cellular phenotypes: loss-of-function mutations abolish NEMO-dependent NF-κB activation and are associated with X-linked dominant Incontinentia Pigmenti (XD-IP) in females and intrauterine death in males (96). Hypoallelic mutations impair but do not abolish NF-κB signaling and are associated with X-linked recessive ectodermal dysplasia with immunodeficiency (XR-EDA-ID) in males (97).In recent years, NEMO exon 5 autoinflammatory syndromes have been reported, resulting from overexpression of NEMO protein isoforms lacking the exon 5 coding domain or non-skewed X-chromosome inactivation (in female carriers) (98, 99). In addition, women carrying heterozygous *NEMO* mutations can also present with immunodeficiency diseases (100, 101).

The immune characteristics of EDA-ID patients are impaired cellular responses of peripheral blood lymphocytes to lipopolysaccharide, IL-1β, IL-18, TNF-α, and CD40 ligand (CD40L) (102). The immune phenotype of patients is highly heterogeneous, and both T and B cell responses may be defective. Patients may present with specific antibody deficiency or decreased lymphocyte proliferation (103). The degree of T cell damage can predict the severity of symptoms in NEMO-deficient patients (104). Excessive type I IFN response, low-density granulocyte dysregulation, and spontaneously activated neutrophils may be the underlying mechanisms of the autoinflammatory phenotype in NEMO-deficient patients (105).Patients are susceptible to invasive pyogenic bacterial infections, including *Streptococcus pneumoniae*, *Haemophilus influenzae, Staphylococcus aureus*, and *Pseudomonas aeruginosa*; nontuberculous mycobacterial infections include *Mycobacterium avium and Mycobacterium kansasii*; severe CMV and HSV infections and fungal infections have also been reported (**Table 1**) (106–108). Ectodermal dysplasia is defined by dental abnormalities, sparse fine hair, frontal bossing, characteristic facies, and decreased sweat gland secretion (109, 110). The features of ectodermal dysplasia vary among patients due to varying penetrance. Other clinical features include osteosclerosis, autoimmunity, arthritis, colitis, lymphadenopathy, ichthyosis, and palmoplantar keratoderma (110–113). These patients require prophylactic treatment with penicillin V and/or trimethoprim-sulfamethoxazole, and, if necessary, immunoglobulin replacement therapy. If the patients’ humoral immune response is normal, vaccinations should be administered as usual. Once infection is suspected, empirical parenteral antibiotic therapy should be initiated immediately, especially for mycobacterial infections, which require aggressive and long-term treatment. Although HSCT cannot correct ectodermal dysplasia or colitis, it can still be included as a method of correcting immunodeficiency (114). A cohort study showed that the median survival rate of children with NEMO deficiency after HSCT was 74% (115).

#### 3.1.12 SPPL2A

*SPPL2A* encodes signal peptide peptidase like 2a (SPPL2a), an intramembrane protease of the GxGD protease family whose substrates include the N-terminal fragment (NTF) of the HLA invariant chain (CD74) expressed by HLA class II^+^ antigen-presenting cells (**Figure 2**) (116). In 2018, three patients with complete AR deficiency were identified from two unrelated consanguineous families from Morocco and Turkey by whole-exome sequencing and genome-wide linkage analysis. Two different homozygous mutations, c.733 + 1G>A and c.1328-1G>A, affecting essential splicing sites, resulted in abnormal mRNA splicing in these patients (**Figure 1**). Overexpression studies demonstrated that the transcripts did not produce protein or produced truncated proteins. CD74 NTF accumulates in HLA class II^+^ myeloid cells and lymphocytes in patients with SPPL2a deficiency. This toxic fragment selectively depletes IL-12 and IL-23-producing CD1c^+^ conventional dendritic cells (cDC2) and their circulating progenitors, resulting in impaired development of type 2 dendritic cells (CD2). In addition, SPPL2a-deficient memory Th1 cells selectively failed to produce IFN-γ when stimulated with mycobacterial antigens *in vitro*. The number and frequency of peripheral T cells in patients were normal, as were the distribution of memory CD4^+^ T cells, γδT cells, and MAIT cell subsets, but the frequency of hematopoietic CD34^+^HLA^-^DR^+^ cells was reduced. The clinical phenotype of these three patients was only that of simple BCG infection, with a good prognosis and no adverse events (**Table 1**) (117). These findings indicate that cDC2 cells are crucial for presenting mycobacterial peptide antigens to CD4^+^ T cells and for the generation of Th1 cells, and that human cDC2 and/or Th1 cells are important for protective immunity against mycobacteria.

#### 3.1.13 TBX21

*TBX21* encodes the transcription factor T-bet, which can bind to the regulatory promoters and enhancers of IFNG and TNF, regulating the production of IFN-γ and TNF-α by human NK and CD4^+^T cells (**Figure 2**) (118). To date, only one patient carrying a homozygous insertion/deletion mutation (c.466_471delGAGATGinsAGTTTA) in the *TBX21* gene has been reported by Yang et al. in 2020 (**Figure 1**). This mutation results in the obstruction of protein production and transport, and destroys the ability of the protein to bind to the common T-box regulatory element in DNA. Due to the lack of transcriptional activation activity, it cannot induce the normal production of IFN-γ in NK and CD4^+^T cells. The patients’ immune phenotype is a significant decrease in the number of NK cells, invariant NKT cells (iNKT), mucosal-associated invariant T cells (MAIT), and Vδ2^+^γδ T lymphocytes. Classical Th1 lymphocytes do not respond to mycobacteria. In summary: T-bet is required for the development of innate cells (NK cells) and innate immune system-like adaptive lymphocytes (iNKT, MAIT, Vδ2^+^γδ T cells) that produce IFN-γ, and this defect cannot be compensated by pure mycobacterium-specific adaptive CD8^+^αβT cells and CCR6^+^CXCR3^+^Th1 cells (119).The patients’ phenotype is disseminated BCG disease, recurrent upper airway hyperresponsiveness, and blood eosinophilia (**Table 1**) (119, 120). The patient responds to antimycobacterial treatment and symptomatic treatment of airway hyperresponsiveness, and the prognosis is good.

### 3.2 Syndromic forms gene

#### 3.2.1 CCR2

CCR2 is the major G protein-coupled receptor for chemokine C-C motif ligand 2 (CCL-2), expressed on human monocytes, dendritic cells, and basophils. It can produce two CCR2 isoforms through alternative splicing (121). Four autosomal recessive homozygous or compound heterozygous mutations in the CCR2 gene were found in nine children from five independent families: p.P214_L215del, p.M61R, p.T296N, p. T21Pfs*18/p. L119R (**Figure 1**). These mutations can affect CCR2 protein expression, CCR2-stimulated calcium ion mobilization, ERK phosphorylation, monocyte migration, and CCL-2 clearance. This defect does not lead to lymphocyte IFN-γ production, GM-CSF signaling, or myeloid host defense dysfunction, suggesting that mycobacterial disease may be caused by impaired CCL2-dependent monocyte recruitment to the site of infection (**Figure 2**). The clinical phenotype of patients with complete AR CCR2 deficiency is mainly manifested in childhood as pulmonary hypertension, significant peri-bronchial and parenchymal lymphocytosis, peribronchiolar pulmonary fibrosis, progressive diffuse parenchymal cyst formation and expansion, progressive obstructive airflow limitation and recurrent secondary infections. Patients are prone to clinically significant infections after vaccination with attenuated live vaccines, showing syndromic MSMD type (9).

#### 3.2.2 ISG15

The *ISG15* (Interferon-Stimulated Gene 15) gene encodes an interferon-induced ubiquitin-like modifier protein that can covalently bind to other proteins, a process also known as ISGylation. ISG15 is present in gelatinase and secretory granules, which release this protein when attacked by bacteria. It is a very effective IFN-γ-inducing cytokine in lymphocytes, especially synergistic with IL-12(**Figure 2**) (122). In 2012, whole exome sequencing first discovered biallelic mutations in ISG15. Currently, fifteen biallelic loss-of-function mutations have been identified, all of which result in autosomal-recessive complete ISG15 deficiency (**Figure 1**) (10, 123–129). Mutations lead to the loss of intracellular ISG15 expression and impaired protein ISGylation. Leukocytes, especially granulocytes, lack the secretion of ISG15 induced by mycobacteria, resulting in reduced IFN-γ secretion by lymphocytes, including natural killer cells (10).Patients with ISG deficiency have an enhanced IFN-α/β response, which is due to the loss of ISG15, which affects the normal accumulation of USP18, a potent negative regulator of IFN-α/β signaling (127, 128). This exaggerated immune response can also be observed in epidermal keratinocytes (128). In addition to recurrent mycobacterial infections, the clinical phenotype of patients can also include neurological manifestations such as intracranial calcification and epilepsy (**Table 1**). Necrotic skin lesions, atopic dermatitis, zinc deficiency, systemic lupus erythematosus, and inflammatory myopathy have also been reported. However, patients do not have adverse reactions to BCG vaccination or viral susceptibility phenotypes (123, 124, 126, 128, 129). Interestingly, based on the pathogenic mechanism of this gene mutation, JAK inhibitors may be an effective cure, and successful cases of treatment with baricitinib and tofacitinib have been reported (123, 130–132). Itaconic acid-based drugs may serve as adjunctive therapy for this rare disease, either (133).

#### 3.2.3 JAK1

The stimulation of IFN-γ signaling can cause the activation of two downstream Janus kinases: JAK1 and JAK2, which in turn leads to the recruitment and phosphorylation of STAT1 protein, driving the expression of genes related to cellular immunity, such as antigen processing and presentation and the activation of bactericidal effector functions(**Figure 2**) (134). JAK1 is also involved in the signal transduction of cytokines of the IL-2, IL-4, IL-7, IL-9, IL-15, IL-21, IL-27, IL-6 family, and IL-10 family. In 2016, the first report of *JAK1* germline mutations was reported, with two homozygous missense P733L and P832S lesions detected in a Pakistani male with a consanguineous marriage (**Figure 1**). The patients’ lymphocytes showed significantly reduced STAT1 phosphorylation after stimulation with IFN-α, IFN-γ, and IL-27. STAT3 phosphorylation was reduced after stimulation with IL-10 (but not IL-6). STAT4 phosphorylation decreased after IFN-α stimulation, STAT5 phosphorylation decreased after IL-2 stimulation, and STAT6 phosphorylation decreased after IL-4 stimulation, suggesting the presence of functional *JAK1* deficiency. There was also a decrease in the induction of interferon-regulated gene expression and dysregulated cytokine production. *In vitro* experiments confirmed that *JAK1* deficiency was mainly attributed to the P733L mutation. The patients’ clinical phenotype was mainly characterized by recurrent systemic non-tuberculous mycobacterium infection (*Mycobacterium malmoense, Mycobacterium scrofulaceum, Mycobacterium gordonae*) (**Table 1**), unexplained cardiomyopathy, developmental delay, and early-onset metastatic bladder cancer. The patient received regular anti-non-tuberculous mycobacterium and anti-cancer treatment and died at the age of 23 due to ineffective chemotherapy. The patients’ IgG level remained high, accompanied by persistent mild T lymphocyte reduction and impaired response to PHA stimulation, with no other obvious abnormalities (135).

#### 3.2.4 RORC

Retinoid-related orphan receptor γt (RORγt) is a member of the nuclear receptor family. It is primarily expressed in the Th17 subset of helper T cells and plays a key role in the differentiation and function of Th17 cells. RORγt regulates the development of thymic T cells and the differentiation of peripheral effector T cells (**Figure 2**). During T cell development, RORγt enhances the survival of CD4^+^CD8^+^ double-positive thymocytes by upregulating the *Bcl-x(L)* gene. In the periphery, RORγt regulates IL-17 production and controls the differentiation of pro-inflammatory Th17, which plays a key role in inflammation and autoimmunity (7, 136). In 2015, Satoshi Okada et al. found biallelic *RORC* LOF mutations in seven individuals from three different ethnic groups with both candidiasis and mycobacterial disease, which were inherited in an autosomal recessive manner (**Figure 1**) (7). The mutations reported so far are S17L, Q308X, and Q420X (**Table 1**). Patients develop the disease in childhood and have a good prognosis, with only one patient dying from disseminated BCG. *RORC*^-/-^ patients exhibit abnormal thymic size and TCRα rearrangement, consistent with mild T-cell lymphopenia, and completely lack MAIT and type 1 NKT cells. The loss of functional RORγ and RORγT isoforms results in a lack of IL-17A/F-producing T cells in these individuals, which may be the cause of their chronic candidiasis. Mycobacterial disease in *RORC*^-/-^ patients may be due to low levels of IFN-γ produced by γδT cells, CCR6^+^CXCR3^+^CD4^+^αβ Th1* cells, or both, after infection with mycobacteria (8).

#### 3.2.5 USP18

The *USP18* gene encodes ubiquitin-specific protease 18 (USP18), an interferon-stimulated gene product and a negative regulator of type I interferon (IFN-I) signaling. It also removes covalently linked ISG15 from proteins through a process called de-ISGylation (**Figure 2**). In turn, ISG15 prevents USP18 from being degraded by the proteasome (137). In 2022, three siblings from the same Moroccan consanguineous family were first reported to carry the homozygous p.I06N mutation, which is a partial USP18 deficiency in AR and manifests as a low-allelic mutation (**Figure 1**). Mutant *USP18* (p.I60N) is normally stabilized by ISG15 and can be efficiently de-ISGylated, but it interacts poorly with receptor-anchored STAT2 and has impaired negative regulation of type I interferon signaling. Because IFN-γ mediates the inability of myeloid cells to produce IL-12 and IL-23, IFN-γ-dependent IL-12 and IL-23 secretion is reduced. Therefore, insufficient negative regulation of IFN-I signaling by *USP18*(p.I60N) is the root cause of a specific type I interferon disease. This mutation will impair the production of IL-12 and IL-23 by myeloid cells, thereby explaining the susceptibility to mycobacterial disease. All three patients developed local fistulizing lymphadenopathy after BCG vaccination. One patient died of systemic inflammation, one patient had central nervous system inflammation and died of excessive inflammation caused by respiratory infection at the age of 9. Only one patient survived to date. Due to the small number of patients reported so far, the optimal treatment for patients with *USP18* deficiency has not yet been determined. The reasons for the inconsistent prognosis of patients remain to be explored, and it is speculated that the abnormal presence of IFN-I (amount, frequency or duration) leads to different pathological outcomes (138).

#### 3.2.6 ZNFX1

Zinc finger NFX1-type containing 1 (ZNFX1) is a highly conserved helicase protein whose potential functions include binding double-stranded RNA to activate antiviral innate immune responses, inhibiting the NLR family pyrin domain-containing protein 3 (NLRP3) inflammasome, and regulating host mRNA stability (**Figure 2**) (139). To date, 27 patients with complete AR *ZNFX1* deficiency have been reported, covering 14 different *ZNFX1* variants (89, 140–142) (**Figure 1**). Some mutations lead to abnormal expression of ZNFX1 protein, but no major defects in IFN-γ production or response have been found in lymphocytes. ZNFX1 is an RNA-binding stress-granule protein essential for monocyte homeostasis and anti-mycobacterial immunity (141). In primary cells from patients with viral infection as the primary phenotype, after stimulation with double-stranded nucleic acids, it was found that mutations cause dysregulated expression patterns of ISGs, altered ISG mRNA half-life, and decreased monocyte viral clearance capacity (140). Clinical phenotypes of patients may include intermittent mononucleosis, mycobacterial disease (BCG or disseminated tuberculosis), severe infections caused by RNA and DNA viruses, viral-induced inflammatory attacks, hemophagocytic syndrome-like diseases, early-onset epilepsy, interstitial pneumonia, and multisystem inflammatory disease (**Table 1**) (140–142). The disease is characterized by a high incidence of hemophagocytic syndrome-like symptoms and a poor prognosis, with more than half of patients dying in childhood (140). Due to limited understanding of the underlying mechanisms of the pathophysiological changes caused by *ZNFX1* mutations, there is currently no effective treatment option. A recent study found that ZNFX1 can inhibit inflammasomes by preventing the translocation of NLRP3, while it is degraded by activated inflammasomes in a feedforward loop. The replenishment of wild-type ZNFX1 protein can rescue the damage of NLRP3, which is of great significance for the development of related treatments (143). However, the specific mechanism of ZNFX1’s effect on the IFN-γ signaling pathway needs further exploration to reveal the underlying causes of the susceptible phenotype of mycobacteria. One study had found that ZNFX1 stabilizes the AMP-activated catalytic subunit α2 mRNA (encoding the essential catalytic subunit α of AMPK) through its zinc finger region, potentially limiting *Mycobacterium tuberculosis* growth (144).

### 3.3 Variable forms genes

#### 3.3.1 IRF8

Interferon regulatory factor 8 (IRF8), also known as interferon consensus sequence binding protein, is one of the nine members of the IRF family of transcription factors. These proteins bind to ISREs and regulate the expression of genes stimulated by IFN-α/β (**Figure 2**). IRF8 is expressed in macrophages and dendritic cells and plays an important role in multiple aspects of myeloid cells (145). *IRF8* deficiency can be classified as complete AR deficiency or partial AD deficiency, and currently only four cases have been reported (**Figure 1**).

Three missense mutations and one compound heterozygous mutation (p.R83C, p.K108E, p.R291Q, c.55del/p.R111*) have been reported in complete AR deficiency (12, 13, 145–147). Severe impairment of IL-12 and IFN-γ induction was observed in PBMCs stimulated with BCG, lectin, or lipopolysaccharide. This immunodeficiency is characterized by a complete absence of CD14^+^ and CD16^+^ circulating monocytes, CD11c^+^ conventional dendritic cells (DCs), and CD11c^+^/CD123^+^ plasmacytoid DCs, while neutrophil counts are elevated. CD4^+^ and CD8^+^ T cell function is reduced, possibly due to a lack of myeloid antigen-presenting cells (13, 147). Patients suffer from a variety of infectious diseases, including disseminated BCG disease, oral candidiasis, and various viral infections such as H1N1 influenza and rhinovirus (**Table 1**). In addition to infections, they may also be accompanied by features such as pulmonary alveolar proteinosis, midface hypoplasia, keratoconus, and brain calcifications (147). In addition to antimicrobial therapy, patients can also receive HSCT as a curative treatment.

The *IRF8* monoallelic mutation (T80A) occurred independently and is not present in the patients’ biological parents or siblings. The T80A mutation is located in the conserved DNA binding domain of *IRF8*, and the T80 residue is strictly conserved among orthologs in all species, showing autosomal dominant inheritance. This mutation mainly causes mild immunodeficiency and causes selective depletion of CD11c^+^CD1c^+^ circulating dendritic cells. Although protein expression is normal, the T80A mutation has pleiotropic effects on IRF8 function, including a significant decrease in DNA binding, which greatly reduces the expression of protein-activated target genes. The mutant allele also has a dominant negative effect on the transcriptional activity of the wild-type protein. The patients’ clinical phenotype is only manifested as disseminated BCG disease, and the prognosis is good without any treatment (13).

#### 3.3.2 STAT1

STAT1 is a transcription factor involved in cellular responses mediated by cytokines such as type I (IFN-α/β), type II (IFN-γ), and type III (IFN-λ) interferons (**Figure 2**) (11). Inherited *STAT1* deficiency comprises four entities: biallelic amorphic, hypomorphic mutations causing AR complete, partial loss-of-function, and monoallelic mutations causing either AD loss-of-function or gain-of-function (148), 76 patients have been reported (**Figure 1**).

Complete AR *STAT1* deficiency has been described in eight mutations in nine patients (11, 149, 150). This mutation is characterized by the absence of wild-type protein expression and loss of cellular responses to antimycobacterial IFN-γ and antiviral IFN-α/β and IFN-λ. Patients’ cells lose GAF and interferon-stimulated gene factor 3 (ISGF3) activity in response to IFN-γ, IFN-α, and IL-27 stimulation (151). After treatment with IFN-α, cells are unable to control viral replication *in vitro*. The clinical phenotype of these patients includes disseminated BCG infection, disseminated nontuberculous mycobacterial infection, and Aspergillus infection, with severe recurrent viral infections (including herpes simplex virus type 1 (HSV-1), CMV, human herpes virus type 8 (HHV-8), varicella-zoster virus vaccine strain, human parainfluenza virus, and RSV). Patients with this pattern of genetic defects have a poor prognosis, with fatal viral or mycobacterial infections reported, and only three patients have successfully undergone HSCT (11, 151, 152).

Some AR *STAT1* deficiencies are caused by biallelic hypomorphic mutations. Since the first report in 2009, six patients have been diagnosed in four families (150, 153–155). The p.P696S, p.K201N, and p.K211R variants have been shown to impair mRNA splicing. STAT1 protein expression levels are approximately 10–30% of wild-type levels, but these residual STAT1 proteins are functional in phosphorylation, dimerization, and DNA binding to target sequences (156). Overall, cells from patients with partial AR *STAT1* deficiency exhibit partially impaired ISGF3 and GAS activity in response to type I and type II IFNs, respectively (153, 156). Based on the residual expression of STAT1, patients have milder clinical symptoms than those with complete AR deficiency. Patients are susceptible to intracellular bacteria (BCG, *Mycobacterium avium, Mycobacterium szulgai, Salmonella*) and viruses (HSV, MCV, RSV and VZV, CMV, COVID-19), and also have phenotypes such as asthma, multiple osteomyelitis, and sinusitis (11, 148, 154). These patients have successfully recovered with anti-infective treatment, with only one death from septic shock.

AD loss-of-function mutations have been reported since 2001, and 20 related mutations have been identified (148, 157–160). Functional experiments have revealed that type II IFN-induced STAT1-mediated signaling is impaired, whereas type I IFN-induced STAT1-mediated signaling is preserved in the context of heterozygous *STAT1* mutations. Although the mutant STAT1 protein is normally expressed, it exerts a dominant-negative effect on type II IFN-induced GAS-mediated signaling, potentially affecting the transcriptional activity of IFN-stimulated response elements and IFN-induced gene expression (158, 159). The severity and underlying mechanisms of loss of function depend on the allele. The most common clinical phenotype in this type of patient is multifocal osteomyelitis, with disseminated *Mycobacterium avium* and BCG infection being the primary infection phenotype. Furthermore, patients typically develop virus-specific antibodies, but clinical penetrance is incomplete (11, 157, 161). The prognosis for patients with this disease is good, with no reported deaths associated with MSMD. Antimicrobial agents and IFN-γ replacement therapy are reliable treatments (158, 159, 162).

#### 3.3.3 TYK2

TYK2 is a member of the Janus Kinase (JAK) family and plays a key role in pathways involving IFN-α/β, IL-6, IL-10, IL-12, and IL-23 responses. It mediates signal transduction through phosphorylation of STAT proteins (**Figure 2**) (163). A total of 43 patients has been reported so far, accounting for 5.20% of all cases. (**Figure 1**)

AR *TYK2* deficiency impairs cellular responses to IL-10, IL-12, IL-23, and IFN-α/β. Impaired responses to IFN-α/β in most cell types underlie viral diseases. Impaired IL-12 and IL-23 responses and Th17 immune deficiency led to chronic mucocutaneous candidiasis in patients. Attenuated IFN-γ induction in IL-12 or IL-23-stimulated lymphocytes underlies mycobacterial diseases (164, 165). In 2006, the first case of AR complete *TYK2* deficiency was reported. The clinical features of the patients were the triad of hyper-IgE syndrome (HIES): atopic dermatitis, high circulating IgE levels, and recurrent cutaneous staphylococcal infections. The patient also suffered from bacterial infections, including lymphadenitis caused by BCG, recurrent gastroenteritis caused by attenuated Mycobacterium Bovis strains, and non-typhoidal Salmonella (165). Subsequently, 25 patients with homozygous *TYK2* mutations were reported. These patients also had viral infections (HSV-1, VZV, influenza A (H1N1) virus, SARS-CoV-2, RSV, EBV, CMV, rubella virus (RV), and JCV virus (John Cunningham virus)) (166). The HIES phenotype was relatively rare (2/26; approximately 8%), and fungal infections were mainly *Candida albicans* (**Table 1**) (164, 167–171).

The clinical phenotype of AR partial deficiency *TYK2* deficiency is mainly mycobacterial infection. The mutations reported so far are R864C, P1104A, G634E, A928V, and G996R (164). These mutations specifically affect the IL-23 pathway. Among them, the homozygous *TYK2* missense mutation P1104A has a low penetrance (possibly <1%), but has a high penetrance (possibly >80%) in tuberculosis in endemic areas. This variant has been shown to affect the enzymatic activity of TYK2, but does not affect its scaffold function or ability to be phosphorylated as a substrate (172, 173).

The overall prognosis of patients with *TYK2* deficiency is good. No patients with fatal viral infection have been found. Most patients are cured but relapse after antimycobacterial treatment. Only one patient died due to ineffective treatment of disseminated extrapulmonary tuberculosis, which may be related to reduced endogenous IFN-γ production. If the patients’ endogenous IFN-γ production is insufficient, exogenous supplementation can be considered, and successful cures have been reported (168, 170). Successful immune reconstitution can be achieved in patients who receive allogeneic HSCT from completely unrelated donors (174).

## 4 Acquired IFN-γ immunodeficiency: anti-IFN-γ autoantibody syndrome (nAIGA)

It is noteworthy that IFN-γ pathway blockade can arise not only from the aforementioned congenital genetic defects but also from acquired autoimmune mechanisms. Neutralizing anti-IFN-γ autoantibody syndrome (nAIGA) represents an acquired, reversible form of blockade of this pathway, forming a continuum of “IFN-γ deficiency” together with hereditary MSMD (175, 176). nAIGA blocks STAT1 phosphorylation downstream of the JAK-STAT pathway by recognizing key IFN-γ epitopes (177). This disrupts the terminal IFN-γ signaling pathway, resulting in susceptibility to intracellular pathogens such as NTM, *Salmonella*, and *Cryptococcus*—clinically mirroring hereditary MSMD (178–180). In stark contrast to hereditary MSMD with childhood onset, nAIGA typically first manifests in adulthood, has no family history, and has a higher incidence in Asian populations (181–183).

In terms of diagnostic strategy, for adult-onset disseminated NTM infections, nAIGA should be ruled out first, followed by consideration of late-onset hereditary MSMD. Treatment differs fundamentally from hereditary MSMD: the latter often requires HSCT for radical cure, while nAIGA, as an acquired disease, can achieve functional cure with B-cell-targeted immunomodulatory therapy (such as rituximab) (184); in some cases, after effective anti-mycobacterial treatment, as the infection is controlled, autoantibody levels can spontaneously and gradually decline (177, 183). This reversible characteristic is a key clinical marker distinguishing it from the lifelong immunodeficiency of hereditary MSMD, suggesting a complex interaction between infection and autoimmunity (177, 182).

## 5 Discussion

Based on the genetic landscape detailed above, the pathogenesis of MSMD can be mechanistically stratified into three hierarchical tiers: IFN-γ production, IFN-γ response, cellular provision and effector execution. These functional layers are interconnected yet distinct, collectively illustrating how genetic defects at discrete nodes trigger cascading failures that ultimately dismantle the host’s anti-mycobacterial defense network (22).

At the IFN-γ production level, defects in the IL-12/23 signaling axis reveal differences in functional redundancy between shared receptor subunits and cell subset-specific receptors. The absence of shared components (such as IL-12Rβ1) simultaneously interferes with multiple cytokines signaling, leading to a general decrease in IFN-γ production; while cytokine receptor defects (*IL12RB2, IL23R*) block IFN-γ production in specific lymphocyte subsets, exhibiting extremely low penetrance due to functional overlap between lymphocyte subsets (67, 87). Transcriptional regulators (*TBX21, RORC*) further control the cell lineage specificity of IFN-γ production by determining the developmental identity of innate-like lymphocytes and Th1 cells (8, 119). Defects in the terminal synthesis and secretion of IFN-γ highlight the importance of strictly regulated post-translational modifications and negative feedback regulation for maintaining IFN-γ production (e.g., *MCTS1, USP18, ISG15*) *(*10, 93, 138).

At the IFN-γ response level, defects in the receptor-signal transduction-transcriptional axis block the target cells’ ability to respond to IFN-γ. Mutations at the receptor level (*IFNGR1, IFNGR2*) produce negative effects through conformational interference, expression defects, or steric hindrance, thereby hindering the formation of functional dimers (25). Allelic mutations in the signal transduction hub *STAT1* have diverse effects that determine the severity of the phenotype (161). Defects in *JAK1*, a shared kinase proximal to the receptor, lead to concurrent impairment of IFN-γ along with multiple signaling pathways such as IL-2, IL-4, and IL-10, resulting in broad-spectrum immunodeficiency (135). Furthermore, the *IRF1* not only mediates the IFN-γ-induced gene expression program but also participates in the early development of effector cells, becoming a dual regulatory node for development and function (91).

At the cellular provision and effector execution level, genetic defects compromise anti-mycobacterial immunity by either depleting the cellular substrates necessary for IFN-γ immunity or incapacitating the terminal effector functions of otherwise intact cells. Defects in genes such as *IRF8, SPPL2A* lead to numerical depletion of specific cell subsets that produce or respond to IFN-γ, such as monocytes, dendritic cells, and innate-like lymphocytes, thereby depriving the immune system of its operative cellular machinery (13, 117)*. CCR2, CYBB* and *ZNFX1* disrupt IFN-γ-mediated anti-mycobacterial immunity by interfering with effector cell recruitment to infection sites, or terminal bactericidal function (9, 19, 141). Taken together, these observations indicate that disturbances in either the quantitative provision of IFN-γ-responsive cells or their qualitative execution of bactericidal functions can be associated with increased susceptibility to mycobacterial disease.

From a mechanistic perspective, the above reveals the functional unity behind the genetic heterogeneity of MSMD. All gene defects point to IFN-γ-centric anti-mycobacterial infection immunity. Notably, the genotype-phenotype association exhibits a significant non-linear characteristic: different genes in the same pathway (such as *IL12RB1* and *IL12RB2*) show drastically different penetrance due to cell type-specific expression and functional redundancy; and different mutations in the same gene determine clinical severity through residual protein function. Furthermore, the discovery of genes such as *USP18*, *ISG15*, and *SPPL2A* has expanded the research scope to new dimensions of protein modification, translation regulation, and organelle quality control. Besides, other immunological disorders might lead to mycobacterium disease like IFN-γ blocking autoantibodies or sometimes no clear genetic component can be identified (e. g. just lower production of IFN-γ) and more investigation regarding such phenotypes are needed.

In terms of diagnosis, targeted panel and NGS has shortened the diagnosis time from months to weeks; however, the pathogenic site has not yet been found in about 50% of families, suggesting that non-classical regulatory regions, somatic chimerism, or dual-gene dose effects have not yet been revealed. The combination of long-read sequencing, single-cell multi-omics, and CRISPR screening is expected to fill this gap and further expand the boundaries of “IFN-γ deficiency diseases”. Current treatments for MSMD include antimycobacterial drug control, adjuvant IFN-γ cytokine replacement therapy, surgical lymph node resection, and HSCT. HSCT is currently the only cure, while gene therapy remains experimental and warrants further exploration. Excitingly, prime editing can achieve high targeted editing activity for gene defects, particularly in patients with p47^phox^-deficient chronic granulomatous disease (185).

In summary, the research focus of MSMD is not only on discovering pathogenic genes to fill 50% of the gaps in understanding, but also on exploring how to achieve early and accurate diagnosis and effective treatment strategies. Effective intervention and treatment to improve the prognosis of MSMD patients is a common goal of all healthcare professionals.

## References

[1] Kerner G Rosain J Guérin A Al-Khabaz A Oleaga-Quintas C Rapaport F . Inherited human IFN-γ deficiency underlies mycobacterial disease. J Clin Invest. (2020) 130:3158–71. doi: 10.1172/jci135460. PMID: 32163377 PMC7260033

[2] Casanova J-L MacMicking JD Nathan CF . Interferon-γ and infectious diseases: Lessons and prospects. Science. (2024) 384:eadl2016. doi: 10.1126/science.adl2016. PMID: 38635718 PMC12539790

[3] Noma K Mizoguchi Y Tsumura M Okada S . Mendelian susceptibility to mycobacterial diseases: state of the art. Clin Microbiol Infection. (2022) 28:1429–34. doi: 10.1016/j.cmi.2022.03.004. PMID: 35283318

[4] Khavandegar A Mahdaviani SA Zaki-Dizaji M Khalili-Moghaddam F Ansari S Alijani S . Genetic, immunologic, and clinical features of 830 patients with Mendelian susceptibility to mycobacterial diseases (MSMD): A systematic review. J Allergy Clin Immunol. (2024) 153:1432–44. doi: 10.1016/j.jaci.2024.01.021. PMID: 38341181 PMC11880893

[5] Philippot Q Ogishi M Bohlen J Puchan J Arias AA Nguyen T . Human IL-23 is essential for IFN-γ-dependent immunity to mycobacteria. Sci Immunol. (2023) 8:eabq5204. doi: 10.1016/j.clim.2023.109481. PMID: 36763636 PMC10069949

[6] Casanova J-L Abel L . The human model: a genetic dissection of immunity to infection in natural conditions. Nat Rev Immunol. (2004) 4:55–66. doi: 10.1038/nri1264. PMID: 14704768

[7] Ogishi M Yang R Rosain J Bustamante J Casanova JL Boisson-Dupuis S . Inborn errors of human transcription factors governing IFN-γ antimycobacterial immunity. Curr Opin Immunol. (2023) 81:102296. doi: 10.1016/j.coi.2023.102296. PMID: 36867972 PMC10023504

[8] Okada S Markle JG Deenick EK Mele F Averbuch D Lagos M . Impairment of immunity to Candida and Mycobacterium in humans with bi-allelic RORC mutations. Science. (2015) 349:606–13. doi: 10.1182/blood.v126.23.205.205 PMC4668938 26160376

[9] Neehus AL Carey B Landekic M Panikulam P Deutsch G Ogishi M . Human inherited CCR2 deficiency underlies progressive polycystic lung disease. Cell. (2024) 187:390–408.e23. doi: 10.1016/j.cell.2023.11.036. PMID: 38157855 PMC10842692

[10] Bogunovic D Byun M Durfee LA Abhyankar A Sanal O Mansouri D . Mycobacterial disease and impaired IFN-γ immunity in humans with inherited ISG15 deficiency. Science. (2012) 337:1684–8. doi: 10.1126/science.1224026. PMID: 22859821 PMC3507439

[11] Boisson-Dupuis S Kong XF Okada S Cypowyj S Puel A Abel L . Inborn errors of human STAT1: allelic heterogeneity governs the diversity of immunological and infectious phenotypes. Curr Opin Immunol. (2012) 24:364–78. doi: 10.1016/j.coi.2012.04.011. PMID: 22651901 PMC3477860

[12] Bigley V Maisuria S Cytlak U Jardine L Care MA Green K . Biallelic interferon regulatory factor 8 mutation: A complex immunodeficiency syndrome with dendritic cell deficiency, monocytopenia, and immune dysregulation. J Allergy Clin Immunol. (2018) 141:2234–48. doi: 10.1016/j.jaci.2017.08.044. PMID: 29128673 PMC5986711

[13] Hambleton S Salem S Bustamante J Bigley V Boisson-Dupuis S Azevedo J . IRF8 mutations and human dendritic-cell immunodeficiency. N Engl J Med. (2011) 365:127–38. doi: 10.1093/med/9780198746690.003.0041. PMID: 21524210 PMC3136554

[14] Ogishi M Arias AA Yang R Han JE Zhang P Rinchai D . Impaired IL-23-dependent induction of IFN-γ underlies mycobacterial disease in patients with inherited TYK2 deficiency. J Exp Med. (2022) 219(10):e20220094. doi: 10.1084/jem.20220094. PMID: 36094518 PMC9472563

[15] Boisson-Dupuis S Ramirez-Alejo N Li Z Patin E Rao G Kerner G . Tuberculosis and impaired IL-23-dependent IFN-γ immunity in humans homozygous for a common TYK2 missense variant. Sci Immunol. (2018) 3(30):eaau8714. doi: 10.1126/sciimmunol.aau8714. PMID: 30578352 PMC6341984

[16] Flannagan RS Jaumouillé V Grinstein S . The cell biology of phagocytosis. Annu Rev Pathol. (2012) 7:61–98. doi: 10.1146/annurev-pathol-011811-132445. PMID: 21910624

[17] Berriozábal-Villarruel X Godínez-Zamora GF Baeza-Capetillo P Pérez-Blanco U Espinosa-Padilla SE Aguirre-Hernández J . Clinical and genetic description of patients with chronic granulomatous disease in a pediatric hospital. Biomedica. (2024) 44:107–17. doi: 10.7705/biomedica.756 PMC12014213 39836837

[18] Nunoi H Nakamura H Nishimura T Matsukura M . Recent topics and advanced therapies in chronic granulomatous disease. Hum Cell. (2023) 36:515–27. doi: 10.1007/s13577-022-00846-7. PMID: 36534309

[19] Bustamante J Arias AA Vogt G Picard C Galicia LB Prando C . Germline CYBB mutations that selectively affect macrophages in kindreds with X-linked predisposition to tuberculous mycobacterial disease. Nat Immunol. (2011) 12:213–21. doi: 10.1038/ni.1992. PMID: 21278736 PMC3097900

[20] Roosens W Wouters M Staels F Gerbaux M Ehlers L Van Loo S . Identification of a novel hypomorphic variant in CYBB underlying an adult presentation of X-linked recessive Mendelian susceptibility to mycobacterial disease. Clin Immunol. (2026) 285:110688. doi: 10.1016/j.clim.2026.110688. PMID: 41765213

[21] Collins HL Kaufmann SH . The many faces of host responses to tuberculosis. Immunology. (2001) 103:1–9. doi: 10.1046/j.1365-2567.2001.01236.x. PMID: 11380686 PMC1783212

[22] Rosain J Kiykim A Michev A Kendir-Demirkol Y Rinchai D Peel JN . Recombinant IFN-γ1b treatment in a patient with inherited IFN-γ Deficiency. J ClinImmunol. (2024) 44:62. doi: 10.1007/s10875-024-01661-5. PMID: 38363432 PMC10873451

[23] Jouanguy E Altare F Lamhamedi-Cherradi S Casanova JL . Infections in IFNGR-1-deficient children. J Interferon Cytokine Res. (1997) 17:583–7. doi: 10.1089/jir.1997.17.583. PMID: 9355958

[24] Olbrich P Martínez-Saavedra MT Perez-Hurtado JM Sanchez C Sanchez B Deswarte C . Diagnostic and therapeutic challenges in a child with complete interferon-γ receptor 1 deficiency. Pediatr Blood Cancer. (2015) 62:2036–9. doi: 10.1002/pbc.25625. PMID: 26173802 PMC4651008

[25] Al-Muhsen S Casanova J-L . The genetic heterogeneity of mendelian susceptibility to mycobacterial diseases. J Allergy Clin Immunol. (2008) 122:1043–51. doi: 10.1016/j.jaci.2008.10.037. PMID: 19084105

[26] Taramasso L Boisson-Dupuis S Garrè ML Bondi E Cama A Nozza P . Pineal germinoma in a child with interferon-γ receptor 1 deficiency. case report and literature review. J ClinImmunol. (2014) 34:922–7. doi: 10.1007/s10875-014-0098-0. PMID: 25216720

[27] Bax HI Freeman AF Anderson VL Vesterhus P Laerum D Pittaluga S . B-cell lymphoma in a patient with complete interferon gamma receptor 1 deficiency. J ClinImmunol. (2013) 33:1062–8. doi: 10.1007/s10875-013-9907-0. PMID: 23800860 PMC3729015

[28] Iyengar VV Chougule A Gowri V Taur P Bodhanwala M Desai MM . Interferon alpha therapy in MSMD. J ClinImmunol. (2024) 44:174. doi: 10.1007/s10875-024-01779-6. PMID: 39120801

[29] Reuter U Roesler J Thiede C Schulz A Classen CF Oelschlagel U . Correction of complete interferon-gamma receptor 1 deficiency by bone marrow transplantation. Blood. (2002) 100:4234–5. doi: 10.1182/blood-2002-02-0433. PMID: 12393576

[30] Rottman M Soudais C Vogt G Renia L Emile JF Decaluwe H . IFN-gamma mediates the rejection of haematopoietic stem cells in IFN-gammaR1-deficient hosts. PloS Med. (2008) 5:e26. doi: 10.1371/journal.pmed.0050026. PMID: 18232731 PMC2214797

[31] Chantrain CF Bruwier A Brichard B Largent V Chapgier A Feinberg J . Successful hematopoietic stem cell transplantation in a child with active disseminated Mycobacterium fortuitum infection and interferon-gamma receptor 1 deficiency. Bone Marrow Transplant. (2006) 38:75–6. doi: 10.1038/sj.bmt.1705399. PMID: 16715106

[32] Dupuis S Döffinger R Picard C Fieschi C Altare F Jouanguy E . Human interferon-gamma-mediated immunity is a genetically controlled continuous trait that determines the outcome of mycobacterial invasion. Immunol Rev. (2000) 178:129–37. doi: 10.1034/j.1600-065x.2000.17810.x. PMID: 11213797

[33] Sologuren I Boisson-Dupuis S Pestano J Vincent QB Fernández-Pérez L Chapgier A . Partial recessive IFN-γR1 deficiency: genetic, immunological and clinical features of 14 patients from 11 kindreds. Hum Mol Genet. (2011) 20:1509–19. doi: 10.1093/hmg/ddr029. PMID: 21266457 PMC3115578

[34] Tomomasa D Tanita K Higashi S Tasaka Y Shimamura T Sakurai U . Early diagnosis of partial interferon-γ receptor 1 deficiency prevents the development of Bacille de Calmette et Guérin osteomyelitis. Clin Immunol. (2022) 235:108933. doi: 10.1016/j.clim.2022.108933. PMID: 35074548

[35] Kong XF Vogt G Chapgier A Lamaze C Bustamante J Prando C . A novel form of cell type-specific partial IFN-gammaR1 deficiency caused by a germ line mutation of the IFNGR1 initiation codon. Hum Mol Genet. (2010) 19:434–44. doi: 10.1093/hmg/ddp507. PMID: 19880857 PMC2800780

[36] Lawrence T Puel A Reichenbach J Ku CL Chapgier A Renner E . Autosomal-dominant primary immunodeficiencies. Curr Opin Hematol. (2005) 12:22–30. doi: 10.1097/01.moh.0000149609.37309.0a. PMID: 15604887

[37] Dorman SE Picard C Lammas D Heyne K van Dissel JT Baretto R . Clinical features of dominant and recessive interferon gamma receptor 1 deficiencies. Lancet. (2004) 364:2113–21. doi: 10.1016/s0140-6736(04)17552-1. PMID: 15589309

[38] Bustamante J Boisson-Dupuis S Abel L Casanova JL . Mendelian susceptibility to mycobacterial disease: genetic, immunological, and clinical features of inborn errors of IFN-γ immunity. Semin Immunol. (2014) 26:454–70. doi: 10.1016/j.smim.2014.09.008. PMID: 25453225 PMC4357480

[39] Alroqi F Almutairi A Alhammadi M Alhamdi S . Successful treatment of invasive mycobacterium infection with interferon beta in a patient with Interferon-Gamma Receptor 1 deficiency. J Infect Public Health. (2024) 17:102468. doi: 10.1016/j.jiph.2024.102468. PMID: 38991411

[40] Vogt G Bustamante J Chapgier A Feinberg J Boisson Dupuis S Picard C . Complementation of a pathogenic IFNGR2 misfolding mutation with modifiers of N-glycosylation. J Exp Med. (2008) 205:1729–37. doi: 10.1016/j.jbiotec.2008.07.389. PMID: 18625743 PMC2525579

[41] Vogt G Chapgier A Yang K Chuzhanova N Feinberg J Fieschi C . Gains of glycosylation comprise an unexpectedly large group of pathogenic mutations. Nat Genet. (2005) 37:692–700. doi: 10.1038/ng1581. PMID: 15924140

[42] Tovo PA Garazzino S Saglio F Scolfaro C Bustamante J Badolato R . Successful hematopoietic stem cell transplantation in a patient with complete IFN-γ Receptor 2 deficiency: a case report and literature review. J ClinImmunol. (2020) 40:1191–5. doi: 10.1007/s10875-020-00855-x. PMID: 32909233 PMC7567729

[43] Indumathi CK Bustamante J . Clinical and immunological profile of children with Mendelian Susceptibility to Mycobacterial Diseases (MSMD) from an Indian tertiary care hospital. Indian J Tuberc. (2021) 68:292–7. doi: 10.1016/j.ijtb.2020.07.027. PMID: 33845970

[44] Peñafiel Vicuña AK Yamazaki Nakashimada M León Lara X Mendieta Flores E Nuñez Núñez ME Lona-Reyes JC . Mendelian susceptibility to mycobacterial disease: retrospective clinical and genetic study in Mexico. J ClinImmunol. (2023) 43:123–35. doi: 10.1007/s10875-022-01357-8 PMC9428379 36044171

[45] Kamoun C Morsheimer M Sullivan KE Holland SM Rundles CC Bunin N . Successful unrelated cord blood transplant for complete IFN-γ receptor 2 deficiency. J Allergy Clin Immunol. (2016) 138:1489–91. doi: 10.1016/j.jaci.2016.06.017. PMID: 27522156

[46] Rosenzweig SD Dorman SE Uzel G Shaw S Scurlock A Brown MR . A novel mutation in IFN-gamma receptor 2 with dominant negative activity: biological consequences of homozygous and heterozygous states. J Immunol. (2004) 173:4000–8. doi: 10.4049/jimmunol.173.6.4000. PMID: 15356149

[47] Toyoda H Ido M Nakanishi K Nakano T Kamiya H Matsumine A . Multiple cutaneous squamous cell carcinomas in a patient with interferon gamma receptor 2 (IFN gamma R2) deficiency. J Med Genet. (2010) 47:631–4. doi: 10.1136/jmg.2009.072108. PMID: 20587411

[48] Rosenzweig SD Schwartz OM Brown MR Leto TL Holland SM . Characterization of a dipeptide motif regulating IFN-gamma receptor 2 plasma membrane accumulation and IFN-gamma responsiveness. J Immunol. (2004) 173:3991–9. doi: 10.4049/jimmunol.173.6.3991. PMID: 15356148

[49] Michniacki TF Walkovich KJ Frame DG Vander Lugt MT . Interferon-γ receptor 1 deficiency corrected by umbilical cord blood transplantation. J ClinImmunol. (2019) 39:257–60. doi: 10.1007/s10875-019-00621-8. PMID: 30953286

[50] Martínez-Barricarte R Megged O Stepensky P Casimir P Moncada-Velez M Averbuch D . Mycobacterium simiae infection in two unrelated patients with different forms of inherited IFN-γR2 deficiency. J ClinImmunol. (2014) 34:904–9. doi: 10.1007/s10875-014-0085-5. PMID: 25135595 PMC4241769

[51] Kilic SS van Wengen A de Paus RA Celebi S Meziane B Hafizoglu D . Severe disseminated mycobacterial infection in a boy with a novel mutation leading to IFN-γR2 deficiency. J Infect. (2012) 65:568–72. doi: 10.1016/j.jinf.2012.08.008. PMID: 22902943

[52] Moncada-Vélez M Martinez-Barricarte R Bogunovic D Kong XF Blancas-Galicia L Tirpan C . Partial IFN-γR2 deficiency is due to protein misfolding and can be rescued by inhibitors of glycosylation. Blood. (2013) 122:2390–401. doi: 10.1182/blood-2013-01-480814. PMID: 23963039 PMC3790508

[53] Oleaga-Quintas C Deswarte C Moncada-Vélez M Metin A Krishna Rao I Kanik-Yüksek S . A purely quantitative form of partial recessive IFN-γR2 deficiency caused by mutations of the initiation or second codon. Hum Mol Genet. (2018) 27:3919–35. doi: 10.1093/hmg/ddy275. PMID: 31222290 PMC6216222

[54] Döffinger R Jouanguy E Dupuis S Fondanèche MC Stephan JL Emile JF . Partial interferon-gamma receptor signaling chain deficiency in a patient with bacille Calmette-Guérin and Mycobacterium abscessus infection. J Infect Dis. (2000) 181:379–84. doi: 10.1086/31519 10608793

[55] Kong XF Vogt G Itan Y Macura-Biegun A Szaflarska A Kowalczyk D . Haploinsufficiency at the human IFNGR2 locus contributes to mycobacterial disease. Hum Mol Genet. (2013) 22:769–81. doi: 10.1093/hmg/dds484. PMID: 23161749 PMC3554203

[56] Rieux-Laucat F Casanova J-L . Autoimmunity by haploinsufficiency. Science. (2014) 345:1560–1. doi: 10.1126/science.1260791. PMID: 25258064

[57] Croxford AL Kulig P Becher B . IL-12-and IL-23 in health and disease. Cytokine Growth Factor Rev. (2014) 25:415–21. doi: 10.1016/j.cytogfr.2014.07.017. PMID: 25130295

[58] Prando C Samarina A Bustamante J Boisson-Dupuis S Cobat A Picard C . Inherited IL-12p40 deficiency: genetic, immunologic, and clinical features of 49 patients from 30 kindreds. Med (Baltimore). (2013) 92:109–22. doi: 10.1097/MD.0b013e31828a01f9 PMC3822760 23429356

[59] Melo KM Tavares FS Antunes TS Condino-Neto A Silva Segundo GR Macedo ACT . Autosomal recessive IL-12p40 deficiency due to a mutation in the IL12B gene: report of a Brazilian patient with lymph node mycobacterial infection. Pediatr Allergy Immunol Pulmonol. (2024) 37:33–6. doi: 10.1089/ped.2022.0206. PMID: 38484269

[60] Sarrafzadeh SA Nourizadeh M Mahloojirad M Fazlollahi MR Shokouhi Shoormasti R Badalzadeh M . Molecular, immunological, and clinical features of 16 Iranian patients with Mendelian susceptibility to mycobacterial disease. J ClinImmunol. (2019) 39:287–97. doi: 10.1007/s10875-019-0593-4. PMID: 30715640

[61] Alodayani AN Al-Otaibi AM Deswarte C Frayha HH Bouaziz M AlHelale M . Mendelian susceptibility to mycobacterial disease caused by a novel founder IL12B mutation in Saudi Arabia. J ClinImmunol. (2018) 38:278–82. doi: 10.1007/s10875-018-0490-2. PMID: 29589181 PMC5934323

[62] Mansouri D Adimi P Mirsaeidi M Mansouri N Khalilzadeh S Masjedi MR . Inherited disorders of the IL-12-IFN-gamma axis in patients with disseminated BCG infection. Eur J Pediatr. (2005) 164:753–7. doi: 10.1007/s00431-005-1689-9. PMID: 16091917

[63] Mahdaviani SA Marjani M Jamee M Khavandegar A Ghaffaripour H Eslamian G . Disseminated Mycobacterium simiae infection in a patient with complete IL-12p40 deficiency. Iran J Allergy Asthma Immunol. (2021) 20:376–81. doi: 10.18502/ijaai.v20i3.6339. PMID: 34134458

[64] Rosenzweig SD Holland SM . Defects in the interferon-gamma and interleukin-12 pathways. Immunol Rev. (2005) 203:38–47. doi: 10.1111/j.0105-2896.2005.00227.x. PMID: 15661020

[65] de Jong R Altare F Haagen IA Elferink DG Boer T van Breda Vriesman PJ . Severe mycobacterial and Salmonella infections in interleukin-12 receptor-deficient patients. Science. (1998) 280:1435–8. doi: 10.1126/science.280.5368.1435. PMID: 9603733

[66] Fieschi C Bosticardo M de Beaucoudrey L Boisson-Dupuis S Feinberg J Santos OF . A novel form of complete IL-12/IL-23 receptor beta1 deficiency with cell surface-expressed nonfunctional receptors. Blood. (2004) 104:2095–101. doi: 10.1182/blood-2004-02-0584. PMID: 15178580

[67] de Beaucoudrey L Samarina A Bustamante J Cobat A Boisson-Dupuis S Feinberg J . Revisiting human IL-12Rβ1 deficiency: a survey of 141 patients from 30 countries. Med (Baltimore). (2010) 89:381–402. doi: 10.1097/MD.0b013e3181fdd832 PMC3129625 21057261

[68] Schepers K Schandené L Bustamante J Van Vooren JP de Suremain M Casanova JL . IL-12Rβ1 deficiency and disseminated Mycobacterium tilburgii disease. J ClinImmunol. (2013) 33:1285–8. doi: 10.1007/s10875-013-9941-y. PMID: 24114017

[69] Esteve-Sole A Sánchez-Dávila SP Deyà-Martínez A Freeman AF Zelazny AM Dekker JP . Severe BCG-osis misdiagnosed as multidrug-resistant tuberculosis in an IL-12Rβ1-deficient Peruvian girl. J ClinImmunol. (2018) 38:712–6. doi: 10.1007/s10875-018-0535-6. PMID: 30039354

[70] Qian M Zhou J Chen P Jiang N Wang T Chen X . A novel compound heterozygous mutation in the IL12RB1 gene causes susceptibility to Mycobacterium tilburgii infection. J ClinImmunol. (2025) 45:133. doi: 10.1007/s10875-025-01930-x. PMID: 41026231 PMC12484313

[71] Fieschi C Dupuis S Catherinot E Feinberg J Bustamante J Breiman A . Low penetrance, broad resistance, and favorable outcome of interleukin 12 receptor beta1 deficiency: medical and immunological implications. J Exp Med. (2003) 197:527–35. doi: 10.1084/jem.20021769. PMID: 12591909 PMC2193866

[72] Uygun H Oren AC Sahinoglu EP Akbayram S . Recurrent visceral leishmaniasis in a case with interleukin-12 receptor beta-1 deficiency. Acta Parasitol. (2024) 69:2069–72. doi: 10.1007/s11686-024-00926-8. PMID: 39388053

[73] Khattak FA Akbar NU Riaz M Hussain M Rehman K Khan SN . Novel IL-12Rβ1 deficiency-mediates recurrent cutaneous leishmaniasis. Int J Infect Dis. (2021) 112:338–45. doi: 10.1016/j.ijid.2021.08.049. PMID: 34438084

[74] León-Lara X Hernández-Nieto L Zamora CV Rodríguez-D'Cid R Gutiérrez MEC Espinosa-Padilla S . Disseminated infectious disease caused by Histoplasma capsulatum in an adult patient as first manifestation of inherited IL-12Rβ1 deficiency. J ClinImmunol. (2020) 40:1051–4. doi: 10.1007/s10875-020-00828-0. PMID: 32710397

[75] Jirapongsananuruk O Luangwedchakarn V Niemela JE Pacharn P Visitsunthorn N Thepthai C . Cryptococcal osteomyelitis in a child with a novel compound mutation of the IL12RB1 gene. Asian Pac J Allergy Immunol. (2012) 30:79–82. 22523911

[76] Vinh DC Schwartz B Hsu AP Miranda DJ Valdez PA Fink D . Interleukin-12 receptor β1 deficiency predisposing to disseminated Coccidioidomycosis. Clin Infect Dis. (2011) 52:e99–e102. doi: 10.1093/cid/ciq215. PMID: 21258095 PMC3060907

[77] Sharifinejad N Mahdaviani SA Jamee M Daneshmandi Z Moniri A Marjani M . Leukocytoclastic vasculitis in patients with IL12B or IL12RB1 deficiency: case report and review of the literature. Pediatr Rheumatol Online J. (2021) 19:121. doi: 10.1186/s12969-021-00623-0. PMID: 34389021 PMC8361607

[78] Chbihi M Boutboul D Berteloot L Casanova JL Bustamante J Lévy R . Salmonella pneumonia in a patient with inherited IL-12Rβ1 deficiency. J ClinImmunol. (2024) 44:132. doi: 10.1007/s10875-024-01722-9. PMID: 38775893

[79] AlKanaan R Almufadhi A Alkahtani RS Alzouman MA Alqubaisy Y Alharthi F . Leukocytoclastic vasculitis in IL-12RB1 deficiency: a rare manifestation of Mendelian susceptibility to mycobacterial disease. Oxf Med Case Rep. (2025) 2025:omaf069. doi: 10.1093/omcr/omaf069. PMID: 40297271 PMC12035693

[80] Arias AA Perez-Velez CM Orrego JC Moncada-Velez M Rojas JL Wilches A . Severe enteropathy and hypogammaglobulinemia complicating refractory Mycobacterium tuberculosis complex disseminated disease in a child with IL-12Rβ1 deficiency. J ClinImmunol. (2017) 37:732–8. doi: 10.1007/s10875-017-0435-1. PMID: 28865061

[81] Luangwedchakarn V Jirapongsaranuruk O NiemeLa JE Thepthai C Chokephaibulkit K Sukpanichnant S . A novel mutation of the IL12RB1 gene in a child with nocardiosis, recurrent salmonellosis and neurofibromatosis type I: first case report from Thailand. Asian Pac J Allergy Immunol. (2009) 27:161–5. 19839503

[82] Ehlayel M de Beaucoudrey L Fike F Nahas SA Feinberg J Casanova JL . Simultaneous presentation of 2 rare hereditary immunodeficiencies: IL-12 receptor beta1 deficiency and ataxia-telangiectasia. J Allergy Clin Immunol. (2008) 122:1217–9. doi: 10.1016/j.jaci.2008.07.005. PMID: 18718650

[83] Tassone L Carvalho AC Calabresi A Tortoli E Apostoli A Scomodon O . Disseminated Mycobacterium genavense infection after immunosuppressive therapy shows underlying new composite heterozygous mutations of β1 subunit of IL-12 receptor gene. J Allergy Clin Immunol. (2013) 131:607–10. doi: 10.1016/j.jaci.2012.05.041. PMID: 22818764

[84] Pourakbari B Hosseinpour Sadeghi R Mahmoudi S Parvaneh N Keshavarz Valian S Mamishi S . Evaluation of interleukin-12 receptor β1 and interferon gamma receptor 1 deficiency in patients with disseminated BCG infection. Allergol Immunopathol (Madr). (2019) 47:38–42. doi: 10.1016/j.aller.2018.06.005. PMID: 30268380

[85] Patel S Uppuluri R Vellaichamy Swaminathan V Ravichandran N Melarcode Ramanan K Raj R . Mendelian susceptibility to mycobacterial disease-challenges in hematopoietic stem cell transplantation. Pediatr Blood Cancer. (2020) 67:e28187. doi: 10.1002/pbc.28187. PMID: 31965686

[86] Pistoia V Cocco C Airoldi I . Interleukin-12 receptor beta2: from cytokine receptor to gatekeeper gene in human B-cell Malignancies. J Clin Oncol. (2009) 27:4809–18. doi: 10.1200/jco.2008.21.3579. PMID: 19720917

[87] Martínez-Barricarte R Markle JG Ma CS Deenick EK Ramírez-Alejo N Mele F . Human IFN-γ immunity to mycobacteria is governed by both IL-12 and IL-23. Sci Immunol. (2018) 3:eaau6759. doi: 10.1126/sciimmunol.aau6759. PMID: 30578351 PMC6380365

[88] Gaffen SL Jain R Garg AV Cua DJ . The IL-23–IL-17 immune axis: from mechanisms to therapeutic testing. Nat Rev Immunol. (2014) 14:585–600. doi: 10.1038/nri3707. PMID: 25145755 PMC4281037

[89] Alawbathani S Westenberger A Ordonez-Herrera N Al-Hilali M Al Hebby H Alabbas F . Biallelic ZNFX1 variants are associated with a spectrum of immuno-hematological abnormalities. Clin Genet. (2022) 101:247–54. doi: 10.1111/cge.14081/v2/response1 34708404

[90] Zhou H Tang YD Zheng C . Revisiting IRF1-mediated antiviral innate immunity. Cytokine Growth Factor Rev. (2022) 64:1–6. doi: 10.1016/j.cytogfr.2022.01.004. PMID: 35090813

[91] Rosain J Neehus A-L Manry J Yang R Le Pen J Daher W . Human IRF1 governs macrophagic IFN-γ immunity to mycobacteria. Cell. (2023) 186:621–645.e33. doi: 10.1016/j.cell.2022.12.038. PMID: 36736301 PMC9907019

[92] Ahmed YL Schleich S Bohlen J Mandel N Simon B Sinning I . DENR-MCTS1 heterodimerization and tRNA recruitment are required for translation reinitiation. PloS Biol. (2018) 16:e2005160. doi: 10.4337/9781035307074.000119 29889857 PMC6013234

[93] Bohlen J Zhou Q Philippot Q Ogishi M Rinchai D Nieminen T . Human MCTS1-dependent translation of JAK2 is essential for IFN-γ immunity to mycobacteria. Cell. (2023) 186:5114–5134.e27. doi: 10.1016/j.cell.2023.09.024. PMID: 37875108 PMC10841658

[94] Zhou Q Bagarić I Komma F Prakash C Abolhassani H Chavoshzadeh Z . Complete and partial forms of X-linked MCTS1 deficiency in patients with mycobacterial disease. J Hum Immun. (2026) 2:e20250073. doi: 10.70962/jhi.20250073. PMID: 41623352 PMC12857535

[95] Legarda-Addison D Hase H O'Donnell MA Ting AT . NEMO/IKKgamma regulates an early NF-kappaB-independent cell-death checkpoint during TNF signaling. Cell Death Differ. (2009) 16:1279–88. doi: 10.1038/cdd.2009.41. PMID: 19373245 PMC2728158

[96] Smahi A Courtois G Vabres P Yamaoka S Heuertz S Munnich A . Genomic rearrangement in NEMO impairs NF-kappaB activation and is a cause of incontinentia pigmenti. The International Incontinentia Pigmenti (IP) Consortium. Nature. (2000) 405:466–72. doi: 10.1038/35013114 10839543

[97] Zonana J Elder ME Schneider LC Orlow SJ Moss C Golabi M . A novel X-linked disorder of immune deficiency and hypohidrotic ectodermal dysplasia is allelic to incontinentia pigmenti and due to mutations in IKK-gamma (NEMO). Am J Hum Genet. (2000) 67:1555–62. doi: 10.1086/316914. PMID: 11047757 PMC1287930

[98] Lee Y Wessel AW Xu J Reinke JG Lee E Kim SM . Genetically programmed alternative splicing of NEMO mediates an autoinflammatory disease phenotype. J Clin Invest. (2022) 132(6):e128808. doi: 10.1172/jci128808. PMID: 35289316 PMC8920334

[99] Eigemann J Janda A Schuetz C Lee-Kirsch MA Schulz A Hoenig M . Non-skewed X-inactivation results in NF-κB essential modulator (NEMO) Δ-exon 5-autoinflammatory syndrome (NEMO-NDAS) in a female with incontinentia pigmenti. J ClinImmunol. (2024) 45:1. doi: 10.1007/s10875-024-01799-2. PMID: 39264518 PMC11393190

[100] Cifaldi C Sgrulletti M Cesare D Rivalta B Emanuele A Colucci L . Partial loss of NEMO function in a female carrier with no incontinentia pigmenti. J Clin Med. (2025) 14(2):363. doi: 10.3390/jcm14020363. PMID: 39860371 PMC11765721

[101] Mou W Zhao Z Gao L Fu L Li J Jiao A . An atypical incontinentia pigmenti female with persistent mucocutaneous hyperinflammation and immunodeficiency caused by a novel germline IKBKG missense mutation. J ClinImmunol. (2023) 43:2165–80. doi: 10.1007/s10875-023-01564-x. PMID: 37831401

[102] Frans G van der Werff Ten Bosch J Moens L Gijsbers R Changi-Ashtiani M Rokni-Zadeh H . Functional evaluation of an IKBKG variant suspected to cause immunodeficiency without ectodermal dysplasia. J ClinImmunol. (2017) 37:801–10. doi: 10.1007/s10875-017-0448-9. PMID: 28993958

[103] Ueki M Hirabayashi S Honda Y Takezaki S Ohata H Abdrabou S . Increased response to granulocyte-macrophage colony-stimulating factor in peripheral blood cells and transient manifestations mimicking juvenile myelomonocytic leukemia in a male patient with NEMO deficiency caused by a deep intronic pathogenic variant of IKBKG. Immunol Med. (2025) 48:94–101. doi: 10.1080/25785826.2024.2422639. PMID: 39485070

[104] Heller S Kölsch U Magg T Krüger R Scheuern A Schneider H . T cell impairment is predictive for a severe clinical course in NEMO deficiency. J ClinImmunol. (2020) 40:421–34. doi: 10.1007/s10875-019-00728-y. PMID: 31965418

[105] Surucu Yilmaz N Bilgic Eltan S Kayaoglu B Geckin B Heredia RJ Sefer AP . Low density granulocytes and dysregulated neutrophils driving autoinflammatory manifestations in NEMO deficiency. J ClinImmunol. (2022) 42:582–96. doi: 10.1007/s10875-021-01176-3. PMID: 35028801

[106] Picard C Casanova JL Puel A . Infectious diseases in patients with IRAK-4, MyD88, NEMO, or IκBα deficiency. Clin Microbiol Rev. (2011) 24:490–7. doi: 10.1128/cmr.00001-11. PMID: 21734245 PMC3131061

[107] Routes J Abinun M Al-Herz W Bustamante J Condino-Neto A De La Morena MT . ICON: the early diagnosis of congenital immunodeficiencies. J ClinImmunol. (2014) 34:398–424. doi: 10.1007/s10875-014-0003-x. PMID: 24619621

[108] Filipe-Santos O Bustamante J Haverkamp MH Vinolo E Ku CL Puel A . X-linked susceptibility to mycobacteria is caused by mutations in NEMO impairing CD40-dependent IL-12 production. J Exp Med. (2006) 203:1745–59. doi: 10.1084/jem.20060085. PMID: 16818673 PMC2118353

[109] Orange JS Jain A Ballas ZK Schneider LC Geha RS Bonilla FA . The presentation and natural history of immunodeficiency caused by nuclear factor kappaB essential modulator mutation. J Allergy Clin Immunol. (2004) 113:725–33. doi: 10.1016/j.jaci.2004.01.762. PMID: 15100680

[110] Kohn LL Braun M Cordoro KM McCalmont TH Shah SD Frieden IJ . Skin and mucosal manifestations in NEMO syndrome: a case series and literature review. Pediatr Dermatol. (2022) 39:84–90. doi: 10.1111/pde.14905. PMID: 34989033

[111] Dupuis-Girod S Corradini N Hadj-Rabia S Fournet JC Faivre L Le Deist F . Osteopetrosis, lymphedema, anhidrotic ectodermal dysplasia, and immunodeficiency in a boy and incontinentia pigmenti in his mother. Pediatrics. (2002) 109:e97. doi: 10.1542/peds.109.6.e97. PMID: 12042591

[112] Ramírez-Alejo N Alcántara-Montiel JC Yamazaki-Nakashimada M Duran-McKinster C Valenzuela-León P Rivas-Larrauri F . Novel hypomorphic mutation in IKBKG impairs NEMO-ubiquitylation causing ectodermal dysplasia, immunodeficiency, incontinentia pigmenti, and immune thrombocytopenic purpura. Clin Immunol. (2015) 160:163–71. doi: 10.1016/j.clim.2015.06.007. PMID: 26117626

[113] Döffinger R Smahi A Bessia C Geissmann F Feinberg J Durandy A . X-linked anhidrotic ectodermal dysplasia with immunodeficiency is caused by impaired NF-kappaB signaling. Nat Genet. (2001) 27:277–85. doi: 10.1038/85837 11242109

[114] Bonilla FA Khan DA Ballas ZK Chinen J Frank MM Hsu JT . Practice parameter for the diagnosis and management of primary immunodeficiency. J Allergy Clin Immunol. (2015) 136:1186–205.e1-78. doi: 10.1016/s1081-1206(10)61142-8. PMID: 26371839

[115] Miot C Imai K Imai C Mancini AJ Kucuk ZY Kawai T . Hematopoietic stem cell transplantation in 29 patients hemizygous for hypomorphic IKBKG/NEMO mutations. Blood. (2017) 130:1456–67. doi: 10.1182/blood-2017-03-771600. PMID: 28679735 PMC5609334

[116] Mentrup T Schröder B . Signal peptide peptidase-like 2 proteases: regulatory switches or proteasome of the membrane? Biochim Biophys Acta Mol Cell Res. (2022) 1869:119163. doi: 10.1016/j.bbamcr.2021.119163. PMID: 34673079

[117] Kong XF Martinez-Barricarte R Kennedy J Mele F Lazarov T Deenick EK . Disruption of an antimycobacterial circuit between dendritic and helper T cells in human SPPL2a deficiency. Nat Immunol. (2018) 19:973–85. doi: 10.1038/s41590-018-0178-z. PMID: 30127434 PMC6130844

[118] Pritchard GH Kedl RM Hunter CA . The evolving role of T-bet in resistance to infection. Nat Rev Immunol. (2019) 19:398–410. doi: 10.1038/s41577-019-0145-4. PMID: 30846856 PMC7272213

[119] Yang R Mele F Worley L Langlais D Rosain J Benhsaien I . Human T-bet governs innate and innate-like adaptive IFN-γ immunity against mycobacteria. Cell. (2020) 183:1826–47.e31. doi: 10.1016/j.cell.2020.10.046. PMID: 33296702 PMC7770098

[120] Yang R Weisshaar M Mele F Benhsaien I Dorgham K Han J . High Th2 cytokine levels and upper airway inflammation in human inherited T-bet deficiency. J Exp Med. (2021) 132(6):e128808. doi: 10.1084/jem.20202726. PMID: 34160550 PMC8225679

[121] Pozzi S Satchi-Fainaro R . The role of CCL2/CCR2 axis in cancer and inflammation: the next frontier in nanomedicine. Adv Drug Delivery Rev. (2024) 209:115318. doi: 10.1016/j.addr.2024.115318. PMID: 38643840

[122] Álvarez E Falqui M Sin L McGrail JP Perdiguero B Coloma R . Unveiling the multifaceted roles of ISG15: from immunomodulation to therapeutic frontiers. Vaccines (Basel). (2024) 12(2):153. doi: 10.3390/vaccines12020153 38400136 PMC10891536

[123] Zhang X Xiong W Ma S Fan S Sun M Zhou Q . A novel homozygous ISG15 missense variant leads to severe inflammatory skin lesions, interstitial pneumonia, and basal ganglia calcifications in a Chinese infant with ISG15 deficiency. Gene. (2025) 960:149537. doi: 10.1016/j.gene.2025.149537. PMID: 40318816

[124] Napoleao S Salgado RC Ferreira JFS de Barros Dorna M de Moura TCL França TT . First Brazilian case report of unrelated patients with identical ISG15 mutation. J ClinImmunol. (2024) 45:21. doi: 10.1007/s10875-024-01811-9. PMID: 39365299

[125] Alzahrani AYB Alghamdi LSA Alghamdi FA . Identification of a novel interferon-stimulated (ISG15) gene variant associated with inflammatory cutaneous lesions and zinc deficiency in a unique family: a case series and literature review. Cureus. (2023) 15:e50701. doi: 10.7759/cureus.50701. PMID: 38234945 PMC10792350

[126] Buda G Valdez RM Biagioli G Olivieri FA Affranchino N Bouso C . Inflammatory cutaneous lesions and pulmonary manifestations in a new patient with autosomal recessive ISG15 deficiency case report. Allergy Asthma Clin Immunol. (2020) 16:77. doi: 10.1186/s13223-020-00473-7. PMID: 32944031 PMC7491304

[127] Zhang X Bogunovic D Payelle-Brogard B Francois-Newton V Speer SD Yuan C . Human intracellular ISG15 prevents interferon-α/β over-amplification and auto-inflammation. Nature. (2015) 517:89–93. doi: 10.1038/nature13801. PMID: 25307056 PMC4303590

[128] Martin-Fernandez M Bravo García-Morato M Gruber C Murias Loza S Malik MNH Alsohime F . Systemic type I IFN inflammation in human ISG15 deficiency leads to necrotizing skin lesions. Cell Rep. (2020) 31:107633. doi: 10.1016/j.celrep.2020.107633. PMID: 32402279 PMC7331931

[129] Al-Mayouf SM Akbar L AlEnazi A Al-Mousa H . Autosomal recessive ISG15 deficiency underlies type I interferonopathy with systemic lupus erythematosus and inflammatory myositis. J ClinImmunol. (2021) 41:1361–4. doi: 10.1007/s10875-021-01019-1. PMID: 33742321

[130] Murias Loza S Courel Del Río V Pardo Campo E Calle-Miguel L Anes González G Rodríguez Suárez J . Successful treatment with tofacitinib in a child diagnosed with ISG15 deficiency. Clin Immunol. (2024) 268:110377. doi: 10.1016/j.clim.2024.110377. PMID: 39401644

[131] Akbar L Alawam B Alhassan M Faisal SY Al-Omari MA Al-Mayouf SM . Case report: ISG15 deficiency and a glimpse into autoimmunity. Int J Rheumatic Dis. (2024) 27:e15420. doi: 10.1111/1756-185x.15420. PMID: 39575596

[132] Burleigh A Moraitis E Al Masroori E Al-Abadi E Hong Y Omoyinmi E . Case report: ISG15 deficiency caused by novel variants in two families and effective treatment with Janus kinase inhibition. Front Immunol. (2023) 14:1287258. doi: 10.3389/fimmu.2023.1287258. PMID: 38115997 PMC10728638

[133] Waqas SF Sohail A Nguyen AHH Usman A Ludwig T Wegner A . ISG15 deficiency features a complex cellular phenotype that responds to treatment with itaconate and derivatives. Clin Transl Med. (2022) 12:e931. doi: 10.1002/ctm2.931. PMID: 35842904 PMC9288839

[134] O'Shea JJ Schwartz DM Villarino AV Gadina M McInnes IB Laurence A . The JAK-STAT pathway: impact on human disease and therapeutic intervention. Annu Rev Med. (2015) 66:311–28. doi: 10.1146/annurev-med-051113-024537 PMC5634336 25587654

[135] Eletto D Burns SO Angulo I Plagnol V Gilmour KC Henriquez F . Biallelic JAK1 mutations in immunodeficient patient with mycobacterial infection. Nat Commun. (2016) 7:13992. doi: 10.1038/ncomms13992. PMID: 28008925 PMC5196432

[136] Sun N Wang Y . RORγt inhibitors in clinical development for the treatment of autoimmune diseases: challenges and opportunities. Expert Opin Ther Pat. (2025) 35(6):583–95. doi: 10.1080/13543776.2025.2482936. PMID: 40110872

[137] Kang JA Jeon YJ . Emerging roles of USP18: from biology to pathophysiology. Int J Mol Sci. (2020) 21(18):6825. doi: 10.3390/ijms21186825. PMID: 32957626 PMC7555095

[138] Martin-Fernandez M Buta S Le Voyer T Li Z Dynesen LT Vuillier F . A partial form of inherited human USP18 deficiency underlies infection and inflammation. J Exp Med. (2022) 219(4):e20211273. doi: 10.1084/jem.20211273. PMID: 35258551 PMC8908790

[139] Cheng LY Parker R . ZNFX1: a multifunctional modulator of the innate immune response. Front Immunol. (2025) 16:1564628. doi: 10.3389/fimmu.2025.1564628. PMID: 40170857 PMC11959080

[140] Vavassori S Chou J Faletti LE Haunerdinger V Opitz L Joset P . Multisystem inflammation and susceptibility to viral infections in human ZNFX1 deficiency. J Allergy Clin Immunol. (2021) 148:381–93. doi: 10.1016/j.jaci.2021.03.045. PMID: 33872655 PMC8569286

[141] Le Voyer T Neehus AL Yang R Ogishi M Rosain J Alroqi F . Inherited deficiency of stress granule ZNFX1 in patients with monocytosis and mycobacterial disease. Proc Natl Acad Sci USA. (2021) 118(15):e2102804118. doi: 10.1073/pnas.2102804118. PMID: 33876776 PMC8053974

[142] Al-Saud B Alshareef T Al-Alwan M Alazami AM . ZNFX1 deficiency in a child with interstitial pneumonitis and peripheral monocytosis. J ClinImmunol. (2023) 43:1529–32. doi: 10.1007/s10875-023-01529-0. PMID: 37291413 PMC10250176

[143] Huang J Wang Y Jia X Zhao C Zhang M Bao M . The human disease-associated gene ZNFX1 controls inflammation through inhibition of the NLRP3 inflammasome. EMBO J. (2024) 43:5469–93. doi: 10.1038/s44318-024-00236-9. PMID: 39333773 PMC11574294

[144] Liu H Han Z Chen L Zhang J Zhang Z Chen Y . ZNFX1 promotes AMPK-mediated autophagy against Mycobacterium tuberculosis by stabilizing Prkaa2 mRNA. JCI Insight. (2024) 9(1):e171850. doi: 10.1172/jci.insight.171850. PMID: 38016036 PMC10906457

[145] Tamura T Yanai H Savitsky D Taniguchi T . The IRF family transcription factors in immunity and oncogenesis. Annu Rev Immunol. (2008) 26:535–47. doi: 10.1146/annurev.immunol.26.021607.090400. PMID: 18303999

[146] Salem S Langlais D Lefebvre F Bourque G Bigley V Haniffa M . Functional characterization of the human dendritic cell immunodeficiency associated with the IRF8(K108E) mutation. Blood. (2014) 124:1894–904. doi: 10.1182/blood-2014-04-570879. PMID: 25122610 PMC4168344

[147] Rosain J Bernasconi A Prieto E Caputi L Le Voyer T Buda G . Pulmonary alveolar proteinosis and multiple infectious diseases in a child with autosomal recessive complete IRF8 deficiency. J ClinImmunol. (2022) 42:975–85. doi: 10.1007/s10875-022-01250-4. PMID: 35338423 PMC8956456

[148] Mizoguchi Y Okada S . Inborn errors of STAT1 immunity. Curr Opin Immunol. (2021) 72:59–64. doi: 10.1016/j.coi.2021.02.009. PMID: 33839590

[149] Sakata S Tsumura M Matsubayashi T Karakawa S Kimura S Tamaura M . Autosomal recessive complete STAT1 deficiency caused by compound heterozygous intronic mutations. Int Immunol. (2020) 32:663–71. doi: 10.1093/intimm/dxaa043. PMID: 32603428

[150] Vairo D Tassone L Tabellini G Tamassia N Gasperini S Bazzoni F . Severe impairment of IFN-γ and IFN-α responses in cells of a patient with a novel STAT1 splicing mutation. Blood. (2011) 118:1806–17. doi: 10.1182/blood-2011-01-330571. PMID: 21772053

[151] Chapgier A Wynn RF Jouanguy E Filipe-Santos O Zhang S Feinberg J . Human complete Stat-1 deficiency is associated with defective type I and II IFN responses *in vitro* but immunity to some low virulence viruses *in vivo*. J Immunol. (2006) 176:5078–83. doi: 10.4049/jimmunol.176.8.5078. PMID: 16585605

[152] Dupuis S Jouanguy E Al-Hajjar S Fieschi C Al-Mohsen IZ Al-Jumaah S . Impaired response to interferon-alpha/beta and lethal viral disease in human STAT1 deficiency. Nat Genet. (2003) 33:388–91. doi: 10.1038/ng1097. PMID: 12590259

[153] Kong XF Ciancanelli M Al-Hajjar S Alsina L Zumwalt T Bustamante J . A novel form of human STAT1 deficiency impairing early but not late responses to interferons. Blood. (2010) 116:5895–906. doi: 10.1182/blood-2010-04-280586. PMID: 20841510 PMC3031383

[154] Kristensen IA Veirum JE Møller BK Christiansen M . Novel STAT1 alleles in a patient with impaired resistance to mycobacteria. J ClinImmunol. (2011) 31:265–71. doi: 10.1007/s10875-010-9480-8. PMID: 21057861

[155] Guèye MS Ndiaye-Diop MT Le Voyer T Soudée C Ba ID Kane A . Inherited STAT1 deficiency in a child with BCG-osis and severe COVID-19 pneumonia. J ClinImmunol. (2023) 43:1479–82. doi: 10.1007/s10875-023-01510-x PMC10232337 37258985

[156] Chapgier A Kong XF Boisson-Dupuis S Jouanguy E Averbuch D Feinberg J . A partial form of recessive STAT1 deficiency in humans. J Clin Invest. (2009) 119:1502–14. doi: 10.1172/jci37083. PMID: 19436109 PMC2689115

[157] Ye F Zhang W Dong J Peng M Fan C Deng W . A novel STAT1 loss-of-function mutation associated with Mendelian susceptibility to mycobacterial disease. Front Cell Infect Microbiol. (2022) 12:1002140. doi: 10.3389/fcimb.2022.1002140. PMID: 36339330 PMC9635896

[158] Chen X Chen J Chen R Mou H Sun G Yang L . Genetic and functional identifying of novel STAT1 loss-of-function mutations in patients with diverse clinical phenotypes. J ClinImmunol. (2022) 42:1778–94. doi: 10.1007/s10875-022-01339-w. PMID: 35976469

[159] Ono R Tsumura M Shima S Matsuda Y Gotoh K Miyata Y . Novel STAT1 variants in Japanese patients with isolated mendelian susceptibility to mycobacterial diseases. J ClinImmunol. (2023) 43:466–78. doi: 10.1007/s10875-022-01396-1. PMID: 36336768

[160] Greybe L Leung D Wieselthaler N le Roux DM Chan KW Lau YL . A rare mutation causing autosomal dominant STAT1 deficiency in a South African multiplex kindred with disseminated BCG infection. BMC Pediatr. (2023) 23:378. doi: 10.1186/s12887-023-04206-8. PMID: 37516851 PMC10386767

[161] Zhang W Chen X Gao G Xing S Zhou L Tang X . Clinical relevance of gain- and loss-of-function germline mutations in STAT1: A systematic review. Front Immunol. (2021) 12:654406. doi: 10.3389/fimmu.2021.654406. PMID: 33777053 PMC7991083

[162] Casanova JL Abel L . Genetic dissection of immunity to mycobacteria: the human model. Annu Rev Immunol. (2002) 20:581–620. doi: 10.1146/annurev.immunol.20.081501.125851. PMID: 11861613

[163] Morand E Merola JF Tanaka Y Gladman D Fleischmann R . TYK2: an emerging therapeutic target in rheumatic disease. Nat Rev Rheumatol. (2024) 20:232–40. doi: 10.1038/s41584-024-01093-w. PMID: 38467779

[164] Ogishi M Arias AA Yang R Han JE Zhang P Rinchai D . Impaired IL-23–dependent induction of IFN-γ underlies mycobacterial disease in patients with inherited TYK2 deficiency. J Exp Med. (2022) 219(10):e20220094. doi: 10.1084/jem.20220094. PMID: 36094518 PMC9472563

[165] Minegishi Y Saito M Morio T Watanabe K Agematsu K Tsuchiya S . Human tyrosine kinase 2 deficiency reveals its requisite roles in multiple cytokine signals involved in innate and acquired immunity. Immunity. (2006) 25:745–57. doi: 10.1016/j.immuni.2006.09.009. PMID: 17088085

[166] Jouanguy E Zhang SY Chapgier A Sancho-Shimizu V Puel A Picard C . Human primary immunodeficiencies of type I interferons. Biochimie. (2007) 89:878–83. doi: 10.1016/j.biochi.2007.04.016. PMID: 17561326

[167] Roussel L Pham-Huy A Yu AC Venkateswaran S Perez A Bourdel G . A novel homozygous mutation causing complete TYK2 deficiency, with severe respiratory viral infections, EBV-driven lymphoma, and jamestown canyon viral encephalitis. J ClinImmunol. (2023) 43:2011–21. doi: 10.1007/s10875-023-01580-x. PMID: 37695435

[168] Kreins AY Ciancanelli MJ Okada S Kong X-F Ramírez-Alejo N Kilic SS . Human TYK2 deficiency: Mycobacterial and viral infections without hyper-IgE syndrome. J Exp Med. (2015) 212:1641–62. doi: 10.1084/jem.20140280. PMID: 26304966 PMC4577846

[169] Lv G Sun G Wu P Du X Zeng T Wen W . Novel mutations of TYK2 leading to divergent clinical phenotypes. Pediatr Allergy Immunol. (2022) 33:e13671. doi: 10.1111/pai.13671. PMID: 34569645

[170] Guo W Feng X Yang M Shangguan Y Shi P Wang S . Mycobacterium intracellulare infection associated with TYK2 deficiency: A case report and review of the literature. Infect Drug Resist. (2020) 13:4347–53. doi: 10.2147/idr.s279438. PMID: 33293838 PMC7719336

[171] Sarrafzadeh SA Mahloojirad M Casanova JL Badalzadeh M Bustamante J Boisson-Dupuis S . A new patient with inherited TYK2 deficiency. J ClinImmunol. (2020) 40:232–5. doi: 10.1007/s10875-019-00713-5. PMID: 31713088 PMC7218688

[172] Boisson-Dupuis S Ramirez-Alejo N Li Z Patin E Rao G Kerner G . Tuberculosis and impaired IL-23–dependent IFN-γ immunity in humans homozygous for a common TYK2 missense variant. Sci Immunol. (2018) 3:eaau8714. doi: 10.1126/sciimmunol.aau8714. PMID: 30578352 PMC6341984

[173] Casanova JL Abel L . From rare disorders of immunity to common determinants of infection: Following the mechanistic thread. Cell. (2022) 185:3086–103. doi: 10.1016/j.cell.2022.07.004. PMID: 35985287 PMC9386946

[174] Gao Y Wang Y Zhou L Lv G Yu J Zhang L . Successful immune reconstitution in a patient with a TYK2 deficiency after allogeneic stem cell transplantation from unrelated donors. J ClinImmunol. (2024) 44:152. doi: 10.1007/s10875-024-01753-2. PMID: 38896258

[175] Browne SK Burbelo PD Chetchotisakd P Suputtamongkol Y Kiertiburanakul S Shaw PA . Adult-onset immunodeficiency in Thailand and Taiwan. N Engl J Med. (2012) 367:725–34. doi: 10.1056/nejmoa1111160. PMID: 22913682 PMC4190026

[176] Jutivorakool K Sittiwattanawong P Kantikosum K Hurst CP Kumtornrut C Asawanonda P . Skin manifestations in patients with adult-onset immunodeficiency due to anti-interferon-gamma autoantibody: A relationship with systemic infections. Acta Derm Venereol. (2018) 98:742–7. doi: 10.2340/00015555-2959. PMID: 29701234

[177] Chen P-K Liao T-L Chang S-H Yeo K-J Chou C-H Chen D-Y . High-titer anti-interferon-γ neutralizing autoantibodies linked to opportunistic infections in patients with adult-onset still's disease. Front Med. (2023) 9:1097514. doi: 10.3389/fmed.2022.1097514. PMID: 36698819 PMC9868624

[178] Chi CY Lin CH Ho MW Ding JY Huang WC Shih HP . Clinical manifestations, course, and outcome of patients with neutralizing anti-interferon-γ autoantibodies and disseminated nontuberculous mycobacterial infections. Med (Baltimore). (2016) 95:e3927. doi: 10.1097/md.0000000000003927. PMID: 27336882 PMC4998320

[179] Wipasa J Chaiwarith R Chawansuntati K Praparattanapan J Rattanathammethee K Supparatpinyo K . Characterization of anti-interferon-γ antibodies in HIV-negative immunodeficient patients infected with unusual intracellular microorganisms. Exp Biol Med (Maywood). (2018) 243:621–6. doi: 10.1177/1535370218764086. PMID: 29512397 PMC6582397

[180] Chetchotisakd P Anunnatsiri S Nithichanon A Lertmemongkolchai G . Cryptococcosis in anti-interferon-gamma autoantibody-positive patients: a different clinical manifestation from HIV-infected patients. Jpn J Infect Dis. (2017) 70:69–74. doi: 10.7883/yoken.jjid.2015.340. PMID: 27169938

[181] Pithukpakorn M Roothumnong E Angkasekwinai N Suktitipat B Assawamakin A Luangwedchakarn V . HLA-DRB1 and HLA-DQB1 are associated with adult-onset immunodeficiency with acquired anti-interferon-gamma autoantibodies. PloS One. (2015) 10:e0128481. doi: 10.1371/journal.pone.0128481. PMID: 26011559 PMC4444022

[182] Patel SY Ding L Brown MR Lantz L Gay T Cohen S . Anti-IFN-gamma autoantibodies in disseminated nontuberculous mycobacterial infections. J Immunol. (2005) 175:4769–76. doi: 10.4049/jimmunol.175.7.4769. PMID: 16177125

[183] Angkasekwinai N Wongsawat E Vorasan N . Clinical course and outcomes of patients with anti-interferon-gamma autoantibody-associated adult-onset immunodeficiency: an observational cohort study. Ann Med. (2025) 57:2565446. doi: 10.1080/07853890.2025.2565446. PMID: 41229127 PMC12616649

[184] Koizumi Y Mikamo H . Anti-interferon gamma autoantibody and disseminated nontuberculous mycobacteria infection: what should be done to improve its clinical outcome? Clin Infect Dis. (2021) 72:2209–11. doi: 10.1093/cid/ciaa1098. PMID: 32745203

[185] Gori JL Haddad E Frangoul H Kohn DB Morris EC Martin BN . Prime editing for p47(phox)-deficient chronic granulomatous disease. N Engl J Med. (2025) 394(12):1195–203. doi: 10.1056/NEJMoa2509807 PMC13533071 41358590

